# Safe and transparent introduction and evaluation of targeted axillary dissection in patients with node-positive breast cancer undergoing primary surgery: international consensus process

**DOI:** 10.1093/bjsopen/zraf121

**Published:** 2025-10-30

**Authors:** Shelley Potter, Ruth Mullan, Henry Cain, Edward R St John, Peter Barry, Yazan Massanat, James Harvey, Katherine Fairhurst, Adrienne Morgan, Margaret Perkins, Gregory Bruce Mann, Jocelyn Lippey, Katherine Cowan, Natalie Blencowe, Stuart A McIntosh, the TADPOLE Trial Management Group and the TADPOLE-TOGETHER Collaborative, Kerry Avery, Petra Baji, Indrani Bhattacharya, Ramsey Cutress, Lucy Culliford, David Dodwell, Jessica Frost, Jessica Harris, Hannah Markham, Elsa Marques, Kirsty Roberts, Sophie Rees, Mohamed Aamer, Nick Abbott, Youmna Abdelaziz, Khaled Abdelwahab, Hussain Abdulla, Belal Abufkhaida, Avi Agrawal, Zarka Ahmad, Mohamed Sherif Ali Ahmed, Shabir Ahmed, Suhair A L Saad, Mahmoud Alhussini, Lolwah Alriyees, Jeha Alshawi, Maila Aslam, Mohamed Attia, Kholoud Awaji, Chwanrow Baban, Abdoul Halim Bagué, Paulina Bajonero-Canonico, Seda Arzuman Baştürk, Faisal Bibal, Efren Bolivar, Maria Bramley, Victoria Brown, Mehmet Zeki Buldanlı, Rita Canotilho, Maria Isabel Carbonell Luna, Amtul R Carmichael, Alexandra Caziuc, Ching Wan Chan, Natalie Chand, Mysore Chandrashekar, Arun Chaubey, Samantha Chen, Iskra Daskalova, Dobromir Dimitrov, Matei Dordea, Stergios Douvetzemis, Mohammad Elahi, Kenneth Elder, Beatrix Elsberger, Sahar Elzein, Angela Fanshawe, Rosa Félix, Clare Fowler, Elena Gallini, Joan Gan, Nidhi Garg, Rares Georgescu, Wafaa Ghazali, Octav Ginghina, Amy R Godden, Omar Gomez-Quiñones, Victoria Graham, Melih Can Gul, Oğuz Hançerlioğulları, Claudia Harding-Mackean, Anita Hargreaves, Grant Harris, Frederick Hartley, James Harvey, Iram Hassan, Saddaqat Hayat, Christopher Holcombe, Jonathan Horsnell, Thomas Hubbard, Alison Hunter-Smith, Rabia Ikram, Tracey Irvine, Gareth Irwin, Jen Isherwood, Arda Isik, Sharmin Islam, Abdulrhman Jaber, Yasmin Jauhari, Marta Jiménez Gómez, Goonj Johri, Charlotte Kallaway, Kemal Erdinc Kamer, Guldeniz Karadeniz Cakmak, Haluk Kerim Karakullukcu, Martin Karamanliev, Isha Karwasra, Vasileios Karydakis, Monika Kaushik, Mah Muneer Khan, Hazem Khout, Stephen Kirk, Cliona Clare Kirwan, Loukiani Kitsikosta, Nurhilal Kiziltoprak, Hannah Knight, Hande Köksal, Petr Krsicka, Katherine Krupa, Jaroslaw Krupa, Gultekin Ozan Kucuk, Risha Lane, Siobhan Laws, Valentina Lefemine, Begonia Lipp Von Wattenwyl, Jocelyn Lippey, Maria Jesús López-Rodriguez, Aoife Lowery, Mhd Zuhri Nanda Rizki Lubis, Emma Macinnes, Draga Mandi, Baris Mantoglu, Patricia Marques Lauria, Lee Martin, Teresa De Jesus Martins Dias, Eleanore Massey, Tahir Masudi, Helen Mathers, Ross Mclean, Ufuk Memiş, Ecem Memişoğlu, Louise Merker, Aikaterini Micha, Ioannis Michalakis, Maria Mir Jan, Geraldine Mitchell, Fatima Mohammed, Radha Raman Mondal, Jenna Morgan, Samantha Muktar, Michelle Mullan, Claire Murphy, Syeda Rifaat Qamar Naqvi, Saman Naz, Harish Neelamraju Lakshmi, Rachel L O'Connell, Enciu Octavian, Bunmi Odofin, Sisse Olsen, Olubukola Omidiji, Hisham Osman, Omer Faruk Ozkan, Lazaros Papadopoulos, Inge Peerlinck, Helena Pereira, Simon Pilgrim, Antonio Piñero-Madrona, Susanna Polotto, Lydia Prusty, Saed Ramzi, Fabio Rapisarda, Rathi Rathinaezhil, Tim Rattay, Jacqueline Rees-Lee, Jaime Alonso Resendiz-Colosia, Catia Ribeiro, Nicola Rocco, Nicola Roche, Fabio Rodrigues, Matthias Rohr, Laszlo Romics, Matthew Rowland, Jennifer Rusby, Sunita Saha, Yasmeen Sajid, Reem Salman, Amtul Shafee Sami, Alejandro Martin Sanchez, Jose I Sanchez-Mendez, Ahmet Necati Sanli, Norah Scally, Lorenzo Scardina, Maria Sciamannini, Natasha Shafique, Lisa Sheehan, Spoorthi Shetty, Meri Shoshkova, Duncan Simpson, Brendan L Skelly, Roslyn Stanton, Nishith Sudhir Mandal, Marios Konstantinos Tasoulis, Amanda Taylor, Natasha Thakur, Rob Thomas, Mangesh Thorat, Amanda Thorne, Dragomir Timbur, Alice Townend, Nora Trabulsi, Hussein Tuffaha, Burak Uçaner, Kalliope Valassiadou, Nikolaos Vassos, Jamie Vatish, Maria Vernet-Tomas, Maria Verroiotou, Rebecca L Wilson, Merve Yesilsancak, Anneza Yiallourou, Ali Cihat Yildirim, Tsanko Yotsov, Charles Zammit, Luis Abraham Zuñiga Vazquez

**Affiliations:** Bristol Surgical and Perioperative Care Complex Intervention Collaboration, Translational Health Sciences, Bristol Medical School, Southmead Hospital, Bristol, UK; Bristol Breast Care Centre, North Bristol NHS Trust, Bristol, UK; Patrick G. Johnston Centre for Cancer Research, Queen’s University Belfast, Belfast, UK; The Newcastle upon Tyne Hospitals NHS Foundation Trust, Newcastle, UK; Portsmouth Hospitals University NHS Trust, Portsmouth, UK; Research Group in Breast Health, University of Portsmouth, Portsmouth, UK; The Royal Marsden NHS Foundation Trust, London, UK; Broomfield Hospital, Mid and South Essex NHS Trust, Broomfield, UK; Manchester University Hospitals NHS Foundation Trust, Manchester, UK; Bristol Surgical and Perioperative Care Complex Intervention Collaboration, Translational Health Sciences, Bristol Medical School, Southmead Hospital, Bristol, UK; The Newcastle upon Tyne Hospitals NHS Foundation Trust, Newcastle, UK; Independent Cancer Patients’ Voice, London, UK; Independent Cancer Patients’ Voice, London, UK; Department of Surgery, The Royal Melbourne Hospital, Melbourne, Victoria, Australia; Department of Surgery, The Royal Women’s Hospital, Melbourne, Victoria, Australia; Breast Cancer Trials, Newcastle, New South Wales, Australia; Department of Surgery, The University of Melbourne, Melbourne, Victoria, Australia; Department of Surgery, The University of Melbourne, Melbourne, Victoria, Australia; Department of Surgery, St. Vincent’s BreastScreen, St. Vincent’s Hospital Melbourne, Melbourne, Victoria, Australia; Department of Surgery, St Vincent’s Hospital Melbourne, Melbourne, Victoria, Australia; Katherine Cowan Consulting, Southend on Sea, UK; Centre for Surgical Research, Population Health Sciences, Bristol Medical School, Bristol, UK; Patrick G. Johnston Centre for Cancer Research, Queen’s University Belfast, Belfast, UK

## Abstract

**Background:**

Axillary node clearance is the current standard of care in patients with node-positive breast cancer undergoing primary surgery, despite a lack of evidence to demonstrate survival benefit and high rates of life-changing morbidity. Targeted axillary dissection (TAD) may be a safe alternative to axillary node clearance, but there is no agreement how primary TAD should be performed. TADPOLE-TOGETHER aimed to use international consensus methods to agree the key components of primary TAD to promote standardized introduction and evaluation of the technique within the TADPOLE trial.

**Methods:**

A scoping review and key stakeholder interviews were used to generate a longlist of possible procedure steps for inclusion in the Delphi questionnaire. Two rounds of an international online survey were then used to agree the mandatory, optional, and prohibited steps of TAD, together with any standardization and training required. The final approach to primary TAD was agreed at an online consensus meeting.

**Results:**

Thirteen potential steps of a TAD procedure were identified from the literature and expert interviews, together with information regarding standardization and training. Some 244 surgeons with global representation participated in the Round 1 survey, of whom 161 (66.0%) participated in Round 2. Seven mandatory steps of primary TAD, including localization and removal of the involved node, combined with a sentinel node biopsy, were agreed upon and ratified by 42 surgeons from the UK, Europe, and Asia who attended the consensus meeting.

**Conclusion:**

Robust international consensus methods have been used to agree how primary TAD should be performed, promoting safe and transparent introduction and evaluation of the technique.

## Teaser

Targeted axillary dissection (TAD) may be an effective alternative to axillary node clearance in patients with node-positive breast cancer undergoing primary surgery. Robust evaluation of primary TAD in a clinical trial is essential, but there are no guidelines regarding how this procedure should be performed. This paper presents the results of an international consensus process in which the surgical approach to primary TAD was agreed upon to support the safe and transparent introduction and evaluation of the technique in the UK TADPOLE trial and beyond, supporting surgeons and protecting patients.

## Introduction

In the UK, the National Institute of Health and Care Excellence currently recommends axillary node clearance (ANC) for all patients with node-positive breast cancer undergoing primary surgery^1^. This highly morbid procedure is recommended to improve local disease control and reduce the risk of breast cancer recurrence, but there is no evidence that ANC improves breast cancer survival, especially in patients with low-volume nodal disease^2–11^. However, one in three patients will experience lifelong complications after ANC, such as lymphoedema (20%)^12^, chronic pain (20%)^13^, or problems with shoulder function, that will significantly affect their long-term quality of life.

Targeted axillary dissection (TAD) is a newer, less invasive technique that may represent a safe alternative to ANC^14^. In the UK and North America, TAD is already recommended in patients with node-positive breast cancer who have a complete response to neoadjuvant systemic therapy (NST)^15,16^, and it may have utility in the primary surgery setting. Targeted removal of the involved lymph node(s) effectively addresses concerns regarding false-negative rates when sentinel node biopsy (SNB) alone is performed in patients with node-positive disease because it ensures the biopsy-proven involved node is removed and facilitates accurate axillary staging to guide adjuvant treatment decision-making^17–20^. However, high-quality evidence from robust randomized clinical trials will be essential to support the introduction of TAD in the primary surgery setting.

The TADPOLE (Targeted Axillary Dissection *versus* Axillary Node Clearance in Patients With Positive Axillary Lymph Nodes in Early Breast Cancer) study is a large-scale multicentre, pragmatic, phase III randomized clinical trial that aims to compare the clinical and cost-effectiveness of TAD and ANC in patients with low-volume node-positive breast cancer having primary surgery. TADPOLE will initially open in the UK in 2025, with plans to open international sites in 2026. However, although TAD in patients undergoing primary surgery (termed primary TAD) is currently offered in a minority of UK centres (∼15%), it is not routinely performed^21^, and a recent scoping review^22^ suggests considerable heterogeneity in how TAD is performed in the neoadjuvant setting.

Therefore, before the TADPOLE trial can commence, it is essential to agree the component steps of primary TAD, whether any of these steps should be performed in a standardized way, and what specific training/experience surgeons wishing to participate in the trial may need. This will form the basis of embedded surgical quality assurance (SQA)^23^ processes within TADPOLE that will ensure that TAD is introduced in a safe and standardized way, is performed consistently in the trial, and can be replicated in wider clinical practice should it be found to be effective. Defining both axillary procedures *a priori* will ensure that TAD and ANC are compared fairly, and the fidelity of both procedures can be effectively monitored as the trial progresses, giving surgeons confidence that axillary surgery in TADPOLE is performed as intended. This will optimize the credibility and acceptability of the trial findings, address many of the challenges of previous surgical trials^23,24^, and increase the likelihood that TADPOLE will be practice changing. It is anticipated that using consensus methods to agree how primary TAD should be performed will optimize ownership of the procedure among the breast surgical community and promote engagement and participation in the trial.

Therefore, TADPOLE-TOGETHER aimed to use robust international consensus methods to agree how primary TAD should be performed to support the safe and transparent introduction and evaluation of the technique in the TADPOLE trial.

## Methods

**Fig. 1:**
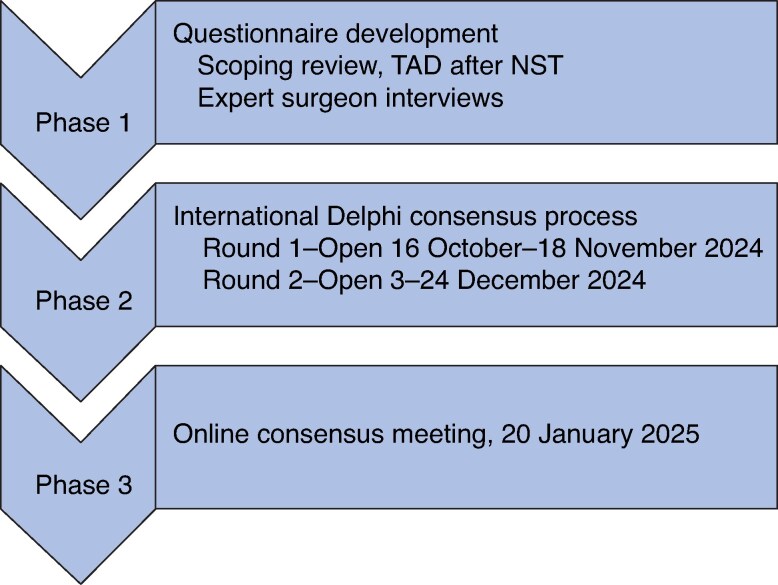
TADPOLE-TOGETHER study flow diagram TAD, targeted axillary dissection; NST, neoadjuvant systemic therapy.

The TADPOLE-TOGETHER project had three phases: phase 1, identification of a comprehensive longlist of potential TAD procedural steps for inclusion in the questionnaire; phase 2, two rounds of an international online Delphi survey with breast cancer surgeons to agree on the mandatory/prohibited steps of a TAD procedure and any standardization and/or training required; and phase 3, an online consensus meeting with expert surgeons to agree and ratify how primary TAD should be performed (*Fig. 1*). The protocol has been published previously^25^, and full ethics approval for the project was obtained from the University of Bristol Faculty of Health Sciences Research Ethics Committee (Reference 20773).

### Phase 1: questionnaire development

#### Generation of a comprehensive list of procedural steps and concomitant interventions for inclusion in the Delphi questionnaire

A scoping review of primary research studies reporting the introduction and/or evaluation of TAD performed in patients after NST was used to identify potential procedural steps for inclusion in the Delphi survey^22^. TAD following NST was chosen because preliminary scoping work failed to identify any research studies reporting TAD performed in patients undergoing primary surgery, and it was considered that the technical steps of a TAD in both settings should be broadly comparable.

Medline and EMBASE were searched from inception up to and including 19 August 2024 using a search strategy developed in collaboration with an expert librarian that included the terms (‘tailored axillary surgery’ OR ‘targeted axillary dissection’ OR ‘selected surgical localization’ OR ‘targeted axillary sampling’ OR ‘targeted axillary surgery’ OR ‘MARI’ (marking axillary lymph nodes with radioactive iodine seeds) OR ‘RISAS’ (Radioactive Iodine Seed localization in the Axilla with the Sentinel node procedure) AND (‘neoadjuvant’). Snowball searching was used to identify any additional relevant papers from the reference lists of identified studies. All primary research studies, published in full, in English, were included and any reported technical step of the procedure was extracted verbatim, together with information regarding the standardization of the procedure and/or training required before performing the technique. Extracted data were categorised using content analysis^26^ by the review team (RM, SP, SAMcI).

Focused interviews with a small group of TAD champions, individuals with expertise in performing primary TAD and/or the introduction and evaluation of novel surgical techniques (HC, EStJ, JH, PB, KF), were used to identify additional relevant procedural steps or considerations when performing primary TAD to ensure the comprehensiveness of the longlist.

#### Delphi survey development

Following review of the longlist and after discussions with the TAD champions, the decision was made to divide the questionnaire into five sections:

- a list of possible procedure steps of primary TAD to be categorised as mandatory (that is, the step must be performed as a component of all primary TAD procedures), optional (that is, performed according to surgeon preference/flexibly), or prohibited (the step must not be performed as part of a primary TAD procedure^24^)
- follow-up questions in which respondents were asked how essential it would be to standardize each step on a nine-point Likert scale from 1 (not essential) to 9 (absolutely essential) based on the Grading of Recommendations Assessment, Development and Evaluation (GRADE) scale^27^ and the proposed approach to standardization informed by the scoping review
- questions regarding the management of difficult situations that may be encountered when performing a primary TAD, and
- for the purposes of the TADPOLE trial, what operative details it would be essential to collect to monitor procedure fidelity in the trial using the nine-point Likert scale described above
- what experience and/or training surgeons who had not performed TAD before would require before starting to perform the procedure.

Respondents were also asked to classify the possible technical steps of an ANC as mandatory, optional, and prohibited to ensure this procedure was also performed consistently within the trial.

Each step was formatted into a questionnaire item. The draft survey was reviewed by the steering group and piloted with a small group of breast surgeons before launch to ensure face and content validity.

### Phase 2: international Delphi survey

Sequential rounds of an international online Delphi survey followed by a consensus meeting were used to agree how primary TAD and ANC should be performed.

#### Sampling and recruitment

Consultant, attending, or equivalent-level surgeons with independent practice and senior trainees (defined as those with a Certificate of Completion of Training (CCT) or within 12 months of their CCT date or equivalent) who routinely treated patients with breast cancer and performed axillary surgery worldwide were invited to participate in the online Delphi survey. The survey was circulated widely through the professional associations, networks, and social media by steering group members.

#### Delphi survey rounds

Participants completed two sequential survey rounds. Round 1 was open between 16 October 2024 and 18 November 2024 and Round 2 was open between 3 and 24 December 2024. The survey was administered online using REDCap^28^ data capture software to facilitate international participation. All participants in Round 1 were invited to participate in Round 2. In Round 2, all questions were accompanied by anonymized feedback about how that item was scored in Round 1, with the aim of promoting consensus across groups whose views may differ^29–31^. Feedback included the participant's own score from Round 1, together with summary scores from all respondents reporting experience of performing TAD completing Round 1. All items were retained between rounds to allow respondents the opportunity to rescore each item in light of the feedback received.

#### Analysis

Simple descriptive statistics were used to summarize respondent demographics in each round.

Following Round 2, the proportion of respondents reporting experience of performing TAD scoring each procedure component as mandatory, optional, or prohibited was calculated, together with the proportions of respondents scoring each standardization item as not essential (scores 1–3), equivocal (scores 4–6), and essential (scores 7–9). The proportions of respondents selecting each standardization method, management strategy, and training requirement were summarized.

Consensus was defined *a priori* as > 70% of respondents expressing agreement regarding mandatory/prohibited steps and other categorical variables. Consensus for standardization and reporting items was defined as > 70% respondents scoring the item very important (7–9), consistent with definitions used in other consensus studies^32–34^. Stata V18 (StataCorp, College Station, TX, USA) was used for all analyses.

#### Attrition

Participant attrition between rounds was monitored and, to optimize participation, regular automatic reminder e-mails were sent to Round 1 participants who had not completed or had only partially completed the Round 2 survey.

### Phase 3: final consensus meeting

The final phase, an online consensus meeting, was held on 20 January 2025. This was initially planned as a face-to-face meeting but converted to a virtual event to facilitate broader engagement and international participation. Surgeons who participated in the Delphi survey and who expressed an interest in attending the consensus meeting were invited to take part. The meeting was facilitated by an independent chair (KC), who encouraged all participants to express their views. All participants were sent a meeting pack before the event that summarized the results of the Delphi survey section by section, with a summary of where consensus had already been achieved and the areas of no consensus where discussion and voting at the meeting would be required.

At the consensus meeting, a summary of the study and consensus process was presented, together with a presentation and discussion of one centre's experience of introducing primary TAD in practice led by one of the TAD champions (HC) for context. The results of each section of the Delphi survey were then discussed in turn, with presentation of consensus in/out items initially and, if disagreements were voiced, a brief facilitated discussion. No consensus items were then presented and discussed. Rounds of anonymous voting using Zoom polls were used to ratify consensus in/out decisions based on Delphi results and establish consensus following discussion for the no consensus items from the Delphi survey.

### Sample size

There is no standard sample size for consensus processes, so the aim was to ensure broad international representation from surgeons with experience performing axillary surgery in patients with breast cancer. It was anticipated that 100–150 surgeons would participate in the Delphi survey and 20–25 would attend the consensus meeting.

## Results

### Phase 1: questionnaire development

Thirteen potential steps of a TAD procedure were identified from the scoping review and expert opinion, together with 11 possible areas where the TAD procedure could be standardized and 10 potential data points that could be collected on case report forms to allow monitoring of procedure fidelity within the future TADPOLE trial. The scoping review identified five difficult situations that may be encountered when performing TAD, and four possible approaches to surgeon credentialing (experience with the procedure and/or training) were devised based on the literature and expert opinion. Items within each section were operationalized into survey items for inclusion in the Delphi survey, a copy of which is provided in *Appendix S1*.

### Phase 2: international Delphi consensus process

In all, 244 surgeons with global representation participated in the Round 1 Delphi survey (*Table 1*). Most respondents (200, 82.0%) were practicing at the consultant level or equivalent, with over half (124, 50.8%) in the early stages of their consultant career (< 10 years experience). Almost 80% of respondents (195, 79.9%) reported having performed at least one TAD procedure, most commonly in the neoadjuvant setting (133, 54.5%), with just over 30% (76, 31%) performing primary TAD in selected patients with low-volume nodal disease and a smaller proportion (37, 15%) reporting performing primary TAD in all patients in this group.

**Table 1:** Delphi participant demographics

	Round 1 (*n* = 244)	Round 2 (*n* = 161)
**Grade**		
Consultant/attending surgeon or equivalent	200 (82%)	136 (85%)
Senior trainee (within 1 year of CCT or post-CCT)	25 (10%)	15 (9%)
Associate specialist	18 (7%)	9 (6%)
Not reported	1 (0%)	0 (0%)
**Years experience as consultant/attending surgeon**		
<5	52 (21%)	34 (21%)
5–10	72 (30%)	52 (32%)
11–15	31 (13%)	19 (12%)
15–20	32 (13%)	19 (12%)
>20	41 (17%)	28 (17%)
Still in training	15 (6%)	8 (5%)
Not reported	1 (0%)	1 (1%)
**Geographical location**		
UK	113 (46%)	82 (51%)
Europe	55 (23%)	36 (22%)
North America	4 (2%)	3 (2%)
Australia/New Zealand	3 (1%)	2 (1%)
Africa	12 (5%)	6 (4%)
Asia	44 (18%)	25 (16%)
Other/not stated	13 (5%)	7 (4%)
**No. of TAD procedures performed to date**		
None	46 (19%)	22 (14%)
<10	62 (25%)	38 (24%)
10–20	57 (23%)	46 (29%)
21–50	42 (17%)	30 (19%)
>50	31 (13%)	20 (12%)
Not stated	6 (3%)	5 (3%)
Local unit practice of TAD*		
Yes—in the neoadjuvant setting	133 (55%)	102 (63%)
Yes—in all patients with low-volume nodal disease (cN0, 1–2 involved nodes on USS) having primary surgery	37 (15%)	21 (13%)
Yes, in selected patients with low-volume nodal disease having primary surgery	76 (31%)	47 (29%)
Not performing TAD in my practice currently	59 (24%)	29 (18%)

Values are *n* (%). *More than one option could be ticked. CCT, Certificate of Completion of Training or equivalent; TAD, targeted axillary dissection; USS, ultrasound scan.

In all, 161 (66.0%) Round 1 participants took part in Round 2. Overall, respondent demographics were similar between rounds, but surgeons with experience performing TADs were more likely to participate in Round 2 (123 of 151, 66.5%) than those without TAD experience (28 of 59, 47.5%; *P* = 0.009, χ^2^ test; *Table 1*).

Following Round 2, consensus was achieved regarding all mandatory, optional, and prohibited steps of both TAD and ANC (*Tables S1*, *S2*), seven TAD standardization items (*Table S1*), and eight case report form items (*Table S3*). Consensus was also achieved on the management of two of the five difficult situations (*Table S4*) and two of the four training/experience requirements (*Table S5*). The remaining items were carried forward for discussion and voting at the consensus meeting.

### Phase 3: consensus meeting

Forty-two consultant surgeons with good international representation (UK: 28, 66.7%; Europe: 9, 21.4%; Asia: 5, 11.9%) and experience performing TAD attended the online consensus meeting. Following discussion and rounds of voting, consensus was reached on how component steps of TAD should be standardized (*Table 2*), which reporting items on case report forms were essential to effectively monitor procedure fidelity in the future TADPOLE trial (*Table S3*), and how difficult situations that may be encountered when performing TAD should be managed (*Table 3*). Although there was broad agreement that there should be no mandated minimum number of nodes that should be removed during primary TAD, during discussions the importance of the ‘denominator’ emerged. Participants highlighted that it was helpful if one of the lymph nodes removed during the procedure was clear (uninvolved) on pathological assessment. Having at least one negative node in the TAD specimen was important in providing reassurance to the multidisciplinary team that more extensive residual axillary disease was unlikely, and further axillary treatment could be omitted.

**Table 2:** Component steps and proposed standardization of a primary TAD procedure

Component steps of a primary TAD	Mandatory or prohibited	Details of how component step is performed	Requirement for standardization of component step
The involved lymph node is localized before surgery	Mandatory	Method for localizing the node	As per local unit practice
		Timing of localization	As per local unit practice
There is confirmation that the involved node is correctly localized before surgery	Mandatory	Method for confirming that the involved node has been correctly localized	As per local unit practice
		Timing of confirmation that the involved node has been correctly localized	As per local unit practice
The skin is marked to demonstrate the position of the localized node before surgery	Optional*	How the skin is marked	As per local unit practice
		When the skin is marked	As per local unit practice
A sentinel node biopsy is performed as part of the primary TAD procedure	Mandatory	Technique used for sentinel node localization	As per local unit practice
		Lymphoscintography is performed	As per local unit practice
		Position of the sentinel node localization injection in the breast	As per local unit practice
		Sentinel node biopsy is performed before removal of the localized node	As per local surgeon practice
The localized node is removed during the TAD procedure	Mandatory	Method for intraoperative confirmation of removal of the localized involved lymph node	As per local unit practice
Removal of the localized node is confirmed during the surgery	Mandatory		
All sentinel nodes are removed during the primary TAD procedure	Mandatory	All sentinel nodes are removed during the primary TAD procedure	
Any palpably abnormal lymph nodes are removed during the primary TAD procedure	Mandatory	Any palpably abnormal lymph nodes are removed during the primary TAD procedure	
A specified minimum number of nodes are removed during primary TAD procedure	Non-mandatory	No specified minimum number of nodes to be removed during a primary TAD procedure	
The total number of lymph nodes that can be removed in the TAD group is limited	Non-mandatory	No specified maximum number of nodes that can be removed during a primary TAD procedure	
Additional normal non-sentinel lymph nodes (not including localized and/or sentinel nodes) are removed	Prohibited	No additional normal non-sentinel lymph nodes should be removed during a primary TAD procedure	
A drain is inserted during the procedure	Optional*	As per local unit practice	
Local anaesthetic is used during the procedure	Optional*	As per local unit practice	

^*^These (optional) steps can be performed or not performed according to local surgeon preference. TAD, targeted axillary dissection.

**Table 3:** Consensus-based management of difficult intraoperative situations that may occur when performing a primary TAD procedure

Situation	Consensus-based management strategy
If the sentinel node localization technique fails (for example, no blue dye/isotope in the axilla) in a patient undergoing primary TAD	Remove the localized involved node and perform a four-node sample (including any palpably abnormal nodes)
If the clip/localization device is identified, but it is not in the involved node in a patient undergoing primary TAD	Perform the SNB and remove any palpably abnormal nodes and any nodes close to the clip/localization device
If the clip/localization device cannot be identified during the procedure in a patient undergoing primary TAD	Perform an SNB, remove any palpably abnormal nodes, and await histology results (further surgery likely to be needed if no involved nodes are identified on pathology)
If neither the sentinel node(s) nor the localized node can be identified during the procedure	Perform a four-node sample including removal of any palpably abnormal nodes
Intraoperative findings are highly suspicious for unanticipated extensive disease in a patient randomized to primary TAD	Remove the localized node, sentinel node, and any palpably abnormal nodes and await histology (patient may require further surgery)

TAD, targeted axillary dissection; SNB, sentinel node biopsy.

Surgeon credentialing (*Table 4*) was discussed at length. Meeting participants strongly felt that it should not be mandatory for surgeons to have either performed TAD in the NST setting or performed a prespecified number of TAD procedures before performing primary TAD in the trial. It was agreed that in addition to engaging with the TADPOLE training, which will include videos and webinars, surgeons wishing to start performing TAD for the first time in the TADPOLE trial should be mentored for their initial cases, and that this mentorship could be facilitated within the trial team.

**Table 4:** Surgeon experience and training to participate in the TADPOLE trial

Recommended training and/or experience	Consensus
Watched TADPOLE training videos and/or attended TADPOLE webinar	Essential
Were familiar with performing TAD in patients following neoadjuvant treatment	Not essential
Had performed a prespecified number of cases	Not essential
Are participating in the ATNEC study	Not essential
Surgeons are mentored for their initial cases if they are performing TAD for the first time*	Essential

^*^Emerged from discussion with expert surgeons at the consensus meeting. TAD, targeted axillary dissection.

## Discussion

TADPOLE-TOGETHER has used robust international consensus methods to agree the technical steps of a primary TAD procedure and how they should (or should not) be standardized and monitored in the future TADPOLE trial. Consensus was achieved on seven mandatory steps, which include localization and removal of the involved lymph node in combination with an SNB in which all the sentinel and palpably abnormal nodes are removed. Confirmation of both correct localization of the involved node before surgery and removal of the localized node during surgery are mandatory steps, and intentional removal of additional normal non-sentinel nodes is prohibited. A very pragmatic approach was taken to procedure standardization, with the consensus view that each step should be performed according to local practice to facilitate widespread participation across multiple units. There was agreement that no prespecified minimum number of nodes should be removed as part of a primary TAD, but the presence of at least one negative node at histology was desirable to provide reassurance that there were not significant amounts of residual axillary disease. Consensus was also achieved regarding the management of difficult situations that may arise when performing a primary TAD, giving surgeons confidence to act based on consensus guidelines should these events occur. Training requirements for surgeons starting to perform TAD were also agreed upon, with mentorship for the first cases essential. Standard criteria for performing an ANC were also agreed. It is anticipated that this work will promote the standardized introduction and robust evaluation of primary TAD in the TADPOLE trial supporting surgeons and protecting patients.

TADPOLE-TOGETHER provides the basis for the SQA within the TADPOLE trial. Quality assurance is a vital component in surgical trials because surgery is a complex intervention that is highly operator dependent. The CONSORT extension for non-pharmacological interventions^35^ mandates the reporting of precise details of both the experimental and comparator intervention, and how adherence to the interventions was assessed or enhanced. Agreeing *a priori* the mandatory and prohibited steps of both axillary procedures will promote standardization of both techniques and allow procedure fidelity to be monitored within the trial. This will give surgeons confidence that the interventions were delivered as intended and the results are credible. A consensus-based approach was specifically chosen to promote engagement and ownership of both the technique and the trial with the breast surgical community and, although embedding radiotherapy quality assurance in breast cancer trials is becoming widely accepted^36–38^, this is the first time SQA has been embedded in a breast cancer surgery trial. This is not surprising because high-quality examples of how to include SQA in surgical trials are lacking^23,39,40^. Failure to include SQA may be one reason why the results of surgical trials are often not accepted and are therefore not implemented into practice^24^. It is anticipated that inclusion of robust radiology, surgical, and radiotherapy quality assurance processes within TADPOLE will optimize the credibility of the trial results and the likelihood that, if positive, it is internationally practice changing.

Despite using robust consensus methods, this work has limitations that require consideration. First, it is possible that not all aspects of primary TAD have been considered and key issues with the technique have been overlooked. Robust longlist development involving a large scoping review and expert surgeon input before questionnaire development suggests that this is unlikely. Furthermore, although the consensus meeting was held online rather than in person, having a highly experienced skilled facilitator and meeting support to monitor the chat in real time led to excellent engagement of meeting participants and extremely productive discussions that elaborated on and contextualised the survey results. Particular attention was paid to variations in practice (for example, sentinel node localization techniques) and ensuring the recommendations were sufficiently flexible and pragmatic so that they could be widely implemented in practice, together with attempting to future proof guidance to allow new devices and/or localization techniques to be used if or when they became available. It is possible that not all surgeons will agree with the proposed recommendations despite the use of robust consensus methods involving over 200 expert surgeons. Therefore, widespread engagement of the breast surgical community through both professional associations and via the influential TAD champions will be essential to optimize the acceptability and adoption of the proposed primary TAD technique. Finally, TADPOLE is a UK-led trial, so the use of international consensus methods to agree how procedures in the trial should be performed may be questioned. Primary TAD is a novel surgical procedure worldwide and there is no consensus how TAD, even in the NST setting, should be performed. Engaging the wider international breast surgical community therefore gives this consensus process utility beyond the TADPOLE trial by agreeing how TAD should be performed more broadly. Engagement was achieved successfully because, although there was limited participation from North American surgeons, as seen in previous studies^41^, there was good representation from surgeons in the UK, Europe, and Asia. Such international engagement in the consensus improves the generalizability of the findings and will support safe and consistent introduction of the technique worldwide.

TADPOLE-TOGETHER has agreed how a primary TAD will be performed, and the next step will be to disseminate these results. The TADPOLE team will now work with the TAD champions and TADPOLE education lead to develop educational materials, including videos and webinars, to support surgeons offering primary TAD within the trial. These resources will be free to access and will promote the safe and transparent introduction and evaluation of the technique within the trial and beyond, including in centres where primary TAD has already been introduced. The consensus-based recommendations will form the basis of the TADPOLE surgical manual, which will accompany the protocol, and the TAD champions will mentor and support surgeons participating in the trial. This, in combination with effective monitoring of procedure fidelity, will ensure that the trial results are credible and meaningful, and, if positive, TADPOLE will provide practice-changing evidence, reducing the morbidity associated with axillary surgery in node-positive breast cancer and improving outcomes for patients.

## Collaborators

The members of the TADPOLE trial management group are: Kerry Avery, Petra Baji, Indrani Bhattacharya, Ramsey Cutress, Lucy Culliford, David Dodwell, Jessica Frost, Jessica Harris, Hannah Markham, Elsa Marques, Kirsty Roberts, and Sophie Rees.

The members of the TADPOLE-TOGETHER Collaborative Group (in alphabetical order) are: Mohamed Aamer (Dr Mohamed Aamer Clinic, Egypt); Nick Abbott (NHS Highland, Inverness, UK); Youmna Abdelaziz (Royal Marsden NHS Trust, London, UK); Khaled Abdelwahab (Mansoura Oncology Center, Mansoura, Egypt); Hussain Abdulla (St Vincent’s University Hospital, Dublin, Ireland); Belal Abufkhaida (Al-Quds University, Jerusalem, Palestine); Avi Agrawal (Portsmouth Hospitals University NHS Trust, UK); Zarka Ahmad (Breast Surgery Unit, Khyber Teaching Hospital, Peshawar, Pakistan); Mohamed Sherif Ali Ahmed (Mansoura University Faculty of Medicine, Mansoura, Egypt); Shabir Ahmed (Mayo Hospital, Lahore, Pakistan); Suhair AL Saad (Dr. Suhair Al Saad Medical Centre, Bahrain); Mahmoud Alhussini (Faculty of Medicine, Alexandria University, Alexandria, Egypt); Lolwah Alriyees (King Fahad Hospital National Guard, King Abdulaziz Medical City, Jeddah, Saudi Arabia); Jeha Alshawi (King Abdulaziz Medical City–National Guard, Jeddah, Saudi Arabia); Maila Aslam (Thq Kharia Doc Hospital Gujrat Mayo Hospital, Lahore, Pakistan); Mohamed Attia (Royal Marsden NHS Trust, London, UK); Kholoud Awaji (King Abdulaziz University Hospital, Jeddah, Saudi Arabia); Chwanrow Baban (University Hospital Limerick, Ireland); Abdoul Halim Bagué (Chu Yalgado Ouedraogo, Ouagadougou, Burkina Faso); Paulina Bajonero-Canonico (Cancer Center Tec 100, Querétaro, Mexico); Seda Arzuman Baştürk (Uşak Training and Research Hospital, Turkey); Faisal Bibal (PUMHS, Nawabshah, Pakistan); Efren Bolivar (Sociedad Venezolana de Mastologia, Caracas, Venezuela); Maria Bramley (Somerset Foundation Trust, Somerset, UK); Victoria Brown (Salisbury Hospital NHS Trust, Salisbury, UK); Mehmet Zeki Buldanlı (University of Health Sciences Gülhane Training and Research Hospital, Ankara, Turkey); Rita Canotilho (Instituto Português De Oncologia Do Porto, Porto, Portugal); Maria Isabel Carbonell Luna (Hospital Quirón Infanta Luisa, Sevilla, Spain); Amtul R. Carmichael (University Hospitals Derby and Burton, UK); Alexandra Caziuc (Iuliu Hatieganu University of Medicine and Pharmacy, Cluj-Napoca, Romania); Ching Wan Chan (Solis Breast Care & Surgery, Singapore); Natalie Chand (Hampshire Hospitals NHS Trust, Hampshire, UK); Mysore Chandrashekar (Royal Liverpool University Hospital, Liverpool, UK); Arun Chaubey (Krishna Surgical Center, Ahmedabad, India); Samantha Chen (Prince of Wales Hospital and The Royal Hospital for Women, Sydney, New South Wales, Australia); Iskra Daskalova (European Medical Center, Moscow, Russia); Dobromir Dimitrov (Centre of Competence in Personalized Medicine, 3D and Telemedicine, Robotic Assisted and Minimally Invasive Surgery, Medical University–Pleven, Pleven, Bulgaria); Matei Dordea (North Tees and Hartlepool Trust, UK); Stergios Douvetzemis (Metropolitan General Hospital of Athens Greece and University of Nicosia Medical School, Greece); Mohammad Elahi (Castle Hill Hospital HUTH, Cottingham, UK); Kenneth Elder (Edinburgh Breast Unit, Edinburgh, UK); Beatrix Elsberger (NHS Grampian, Aberdeen Royal Infirmary, Aberdeen, UK); Sahar Elzein (Mid Cheshire Hospitals NHS Foundation Trust, Cheshire, UK); Angela Fanshawe (London North West University Healthcare NHS Trust, London, UK); Rosa Félix (Hospital Espírito Santo Évora, Évora, Portugal); Clare Fowler (Gloucestershire Hospitals NHS Foundation Trust, UK); Elena Gallini (Kingston and Richmond Hospital NHS Foundation Trust, UK); Joan Gan (Regency Specialist Hospital, Johor Darul Ta'zim, Malaysia); Nidhi Garg (Whipps Cross University Hospital, London, UK); Rares Georgescu (Mureş County Hospital, Romania); Wafaa Ghazali (University of Jeddah, Jeddah, Saudi Arabia); Octav Ginghina (Oncology Institute of Bucharest, Bucharest, Romania); Amy R. Godden (Royal Marsden NHS Trust, London, UK); Omar Gomez-Quiñones (Instituto Mexicano Del Seguro Social, Juárez, Mexico); Victoria Graham (Altnagelvin Hospital, Derry, UK); Melih Can Gul (Afyonkarahisar State Hospital General Surgery Department, Afyonkarahisar, Turkey); Oğuz Hançerlioğulları (University of Health Sciences, Gülhane Training and Research Hospital, Ankara, Turkey); Claudia Harding-Mackean (Countess of Chester NHS Foundation Trust, Chester, UK); Anita Hargreaves (Countess of Chester NHS Foundation Trust, Chester, UK); Grant Harris (Northumbria Healthcare NHS Foundation Trust, UK); Frederick Hartley (Royal Marsden NHS Trust, London, UK); James Harvey (University of Manchester, Manchester, UK); Iram Hassan (University Hospital Southampton, Southampton, UK); Saddaqat Hayat (Dr Faisal Masood Teaching Hospitals, Sargodha, Pakistan); Christopher Holcombe (Swansea Bay University Health Board, Swansea, UK); Jonathan Horsnell (Royal Surrey NHS Foundation Trust, UK); Thomas Hubbard (University of Exeter, Exeter UK); Alison Hunter-Smith (Royal United Hospital, Bath, UK); Rabia Ikram (Shaikh Zayed Hospital and Rashid Latif Medical Complex, Lahore, Pakistan); Tracey Irvine (Royal Surrey NHS Foundation Trust, UK); Gareth Irwin (Belfast Health and Social Care Trust, UK); Jen Isherwood (Milton Keynes University Trust, Milton Keynes, UK); Arda Isik (Istanbul Medeniyet University, Istanbul, Turkey); Sharmin Islam (Dhaka Medical College Hospital, Dhaka, Bangladesh); Abdulrhman Jaber (Zliten Medical Centre, Zliten, Libya); Yasmin Jauhari (Southampton University Hospital NHS Trust, UK); Marta Jiménez Gómez (Hospital Del Mar, Barcelona, Spain); Goonj Johri (Princess Anne Hospital, University Hospital Southampton, Southampton, UK); Charlotte Kallaway (Gloucestershire Hospitals NHS Foundation Trust, UK); Kemal Erdinc Kamer (Izmir City Hospital, Izmir, Turkey); Guldeniz Karadeniz Cakmak (Zonguldak Bulent Ecevit University, The School of Medicine Breast Center, Zonguldak, Turkey); Haluk Kerim Karakullukcu (Sultan 2, Abdulhamid Khan Training and Research Hospital, Istanbul, Turkey); Martin Karamanliev (Centre of Competence in Personalized Medicine, 3D and Telemedicine, Robotic Assisted and Minimally Invasive Surgery, Medical University-Pleven, Pleven, Bulgaria); Isha Karwasra (Princess Anne Hospital, University Hospital Southampton, Southampton, UK); Vasileios Karydakis (Guy’s & St Thomas’ NHS Foundation Trust and Medway NHS Foundation Trust, UK); Monika Kaushik (University Hospitals of Leicester NHS Trust, Leicester UK); Mah Muneer Khan (Khyber Medical College and Khyber Teaching Hospital, Peshawar, Pakistan); Hazem Khout (Nottingham Breast Institute, Nottingham, UK); Stephen Kirk (The South Eastern Trust, UK); Cliona Clare Kirwan (University of Manchester, Manchester, UK); Loukiani Kitsikosta (University Hospital Southampton, Southampton, UK); Nurhilal Kiziltoprak (Sultan Abdulhamid Han Research and Training Hospital, Istanbul, Turkey); Hannah Knight (Royal Devon University Healthcare Foundation Trust, Exeter, UK); Hande Köksal (Selcuk University, Faculty of Medicine, General Surgery Clinic, Konya, Turkey); Petr Krsicka (Masaryk Memorial Cancer Institute, Brno, Czech Republic); Katherine Krupa (Royal Marsden NHS Trust, London, UK); Jaroslaw Krupa (University Hospitals of Leicester NHS Trust, Leicester, UK); Gultekin Ozan Kucuk (Samsun Training and Research Hospital, İlkadım/Samsun, Turkey); Risha Lane (Ipswich Hospital, ESNEFT, UK); Siobhan Laws (Hampshire Hospitals NHS Trust, Hampshire, UK); Valentina Lefemine (Aneurin Bevan University Health Board, UK); Begonia Lipp Von Wattenwyl (Regional Hospital of Bellinzona, Bellinzona, Switzerland); Jocelyn Lippey (St. Vincent’s Hospital, Melbourne, Victoria, Australia); Maria Jesús López-Rodriguez (Hospital Universitario Ramón y Cajal, Madrid, Spain); Aoife Lowery (University of Galway, Gallway, Ireland ); Mhd Zuhri Nanda Rizki Lubis (Peraboi, Indonesia); Emma Macinnes (Leeds Teaching Hospitals, UK); Draga Mandi (Coltea Clinical Hospital, Bucharest, Romania); Baris Mantoglu (Sakarya University, Faculty of Medicine Department of General Surgery, Sakarya, Turkey); Patricia Marques Lauria (Clinical Hospital—Federal University, Belo Horizonte, Brazil); Lee Martin (Liverpool University Foundation Trust, Liverpool, UK); Teresa De Jesus Martins Dias (Instituto Português De Oncologia Do Porto, Porto, Portugal); Eleanore Massey (Gloucestershire Hospitals NHS Foundation Trust, UK); Tahir Masudi (Rotherham Foundation Trust Hospital, UK); Helen Mathers (Southern Health and Social Care Trust, UK); Ross Mclean (Queen Elizabeth Hospital, Gateshead, UK); Ufuk Memiş (Erzincan Binali Yıldırım Üniversitesi Tıp Fakültesi, Erzincan, Turkey); Ecem Memişoğlu (Kartal Dr Lütfi Kırdar City Hospital, Istanbul, Turkey); Louise Merker (Torbay and South Devon NHS Foundation Trust, UK); Aikaterini Micha (Kingston Hospital NHS Trust, UK); Ioannis Michalakis (North Cumbria Integrated Care NHS Foundation Trust, UK); Maria Mir Jan (Fauji Foundation Hospital/Foundation Medical University, Islamabad, Pakistan); Geraldine Mitchell (Liverpool University Foundation Trust, Liverpool, UK); Fatima Mohammed (Omdurman Islamic University, Omdurman, Sudan); Radha Raman Mondal (Medical College and Hospital, Kolkata, India); Jenna Morgan (University of Sheffield, Sheffield, UK); Samantha Muktar (London North West University Healthcare Trust, UK); Michelle Mullan (North Bristol NHS Trust, Bristol, UK); Claire Murphy (Airedale NHS Trust, UK); Syeda Rifaat Qamar Naqvi (Combined Military Hospital, Rawalpindi, Pakistan); Saman Naz (Health Department, Pakistan); Harish Neelamraju Lakshmi (Yashoda Hospitals Hitec City, Hyderabad, India); Rachel L. O'Connell (Royal Marsden NHS Trust, London, UK); Enciu Octavian (Elias University Emergency Hospital, Bucharest, Romania); Bunmi Odofin (University Hospital Southampton, Southampton, UK); Sisse Olsen (Royal Devon University Healthcare Foundation Trust, Exeter, UK); Olubukola Omidiji (Plymouth); Hisham Osman (Frimley Park Hospital, Frimley, UK); Omer Faruk Ozkan (University of Health and Science, Abdulhamid Training and Research Hospital, Istanbul, Turkey); Lazaros Papadopoulos (Breast Unit, Iaso Hospital, Athens Greece); Inge Peerlinck (East Suffolk and North Essex Foundation Trust, UK); Helena Pereira (Instituto Português De Oncologia Do Porto, Porto, Portugal); Simon Pilgrim (University Hospitals of Leicester NHS Trust, Leicester, UK); Antonio Piñero-Madrona (Hospital Clínico Universitario ‘Virgen De La Arrixaca’, Murcia, Spain); Susanna Polotto (Nottingham Breast Institute, Nottingham, UK); Lydia Prusty (Lincoln County Hospital, United Lincolnshire NHS Trust, UK); Saed Ramzi (University Hospitals Plymouth NHS Trust, Plymouth, UK); Fabio Rapisarda (East Suffolk and North Essex Foundation Trust, UK); Rathi Rathinaezhil (University Hospital Sussex NHS Trust, UK); Tim Rattay (University of Leicester, Leicester, UK); Jacqueline Rees-Lee (Torbay and South Devon NHS Foundation Trust, UK); Jaime Alonso Resendiz-Colosia (Instituto Mexicano Del Seguro Social, Juárez, Mecico); Catia Ribeiro (Instituto Português De Oncologia Do Porto, Porto, Portugal); Nicola Rocco (University of Naples Federico II, Naples, Italy); Nicola Roche (Royal Marsden NHS Trust, London, UK); Fabio Rodrigues (Instituto De Câncer Dr Arnaldo/Centro De Oncologia E Hematologia Beneficência Portuguesa, São Paulo, Brazil); Matthias Rohr (Intermediate Hospital, Windhoek, Namibia); Laszlo Romics (Gartnavel General Hospital, Glasgow, NHS GGC, UK); Matthew Rowland (Liverpool University Foundation Trust, Liverpool, UK); Jennifer Rusby (Royal Marsden NHS Trust, London, UK); Sunita Saha (Colchester Hospital, East Suffolk and North Essex NHS Foundation Trust, Colchester, UK); Yasmeen Sajid (Gujranwala Medical Complex, Gujranwala, Pakistan); Reem Salman (Beaumont Hospital Dublin, Ireland); Amtul Shafee Sami (Lincoln County Hospital, United Lincolnshire NHS Trust, UK); Alejandro Martin Sanchez (Multidisciplinary Breast Center—Dipartimento Scienze Della Salute Della Donna E Del Bambino E Di Sanità Pubblica, Fondazione Policlinico Universitario A. Gemelli IRCCS, Rome, Italy); Jose I. Sanchez-Mendez (Hospital Universitario La Paz, Madrid, Spain); Ahmet Necati Sanli (Abdulkadir Yuksel State Hospital, Gaziantep, Turkey); Norah Scally (Craigavon Area Hospital, Craigavon, UK); Lorenzo Scardina (Fondazione Policlinico Universitario Agostino Gemelli IRCCS, Rome, Italy); Maria Sciamannini (Breast Unit Ospedale San Jacopo, Pistoia, Italy); Natasha Shafique (Sherwood Forest Hospital Trust, UK); Lisa Sheehan (Plymouth University Hospitals, Plymouth); Spoorthi Shetty (Maidstone Tunbridge Wells Hospital, UK); Meri Shoshkova (Medical University–Pleven, Pleven, Bulgaria); Duncan Simpson (Antrim Area Hospital, Antrim, UK); Brendan L. Skelly (Western HSCT, UK); Roslyn Stanton (Hampshire Hospitals NHS Trust, Hampshire, UK); Nishith Sudhir Mandal (Vardhman Mahavir Medical College and Safdarjung Hospital, Delhi, India); Marios Konstantinos Tasoulis (The Royal Marsden NHS Foundation Trust, UK); Amanda Taylor (Milton Keynes University Trust, Milton Keynes, UK); Natasha Thakur (SKIMS Institute of Medical Sciences, Kashmir, India); Rob Thomas (Newcastle upon Tyne Hospitals NHS Trust, Newcastle, UK); Mangesh Thorat (Centre for Cancer Screening, Prevention and Early Diagnosis, Wolfson Institute of Population Health, Queen Mary University of London, London, UK); Amanda Thorne (Musgrove Park Breast Unit, Taunton, UK); Dragomir Timbur (Medpark International Hospital, Chişinău, Moldova); Alice Townend (Northumbria Healthcare NHS Foundation Trust, UK); Nora Trabulsi (King Abdulaziz University Hospital, Jeddah, Saudi Arabia); Hussein Tuffaha (East Suffolk and North Essex Foundation Trust, UK); Burak Uçaner (University of Health Sciences, Gülhane Training and Research Hospital, Ankara, Turkey); Kalliope Valassiadou (University Hospitals of Leicester NHS Trust, Leicester, UK); Nikolaos Vassos (Department of Surgical Oncology, Athens Medical Center, Athens, Greece); Jamie Vatish (University Hospitals Birmingham, Birmingham, UK); Maria Vernet-Tomas (Hospital Del Mar, Barcelona, Spain); Maria Verroiotou (University Hospitals, Plymouth, UK); Rebecca L. Wilson (University Hospital of North Tees and Hartlepool, UK); Merve Yesilsancak (Sakarya Training and Research Hospital (Sakarya Egitim Arastirma Hastanesi, Adapazarı/Sakarya, Turkey); Anneza Yiallourou (Medical School, University of Cyprus, Nicosia, Cyprus); Ali Cihat Yildirim (Kütahya Health Sciences University, Kütahya, Turkey); Tsanko Yotsov (Ruse University Angel Kanchev, UMHAT Medica, Ruse, Bulgaria); Charles Zammit (Brighton Breast Unit–Universities Sussex Foundation Trust, UK); Luis Abraham Zuñiga Vazquez (Hospital Aranda De La Parra, León, Mexico).

## Supplementary Material

zraf121_Supplementary_Data

## Data Availability

All data from this study are presented in the paper, tables, and supplementary materials. No further data are available.

## References

[1] National Institute for Heath and Care Excellence (NICE) . *Early and locally advanced breast cancer: diagnosis and management NICE guideline [NG101]*. https://www.nice.org.uk/guidance/ng101 (accessed 22 August 2025) 35263062

[2] Galimberti V, Cole BF, Viale G, Veronesi P, Vicini E, Intra M et al Axillary dissection *versus* no axillary dissection in patients with breast cancer and sentinel-node micrometastases (IBCSG 23-01): 10-year follow-up of a randomised, controlled phase 3 trial. Lancet Oncol 2018;19:1385–1393 30196031 10.1016/S1470-2045(18)30380-2

[3] Galimberti V, Cole BF, Zurrida S, Viale G, Luini A, Veronesi P et al Axillary dissection *versus* no axillary dissection in patients with sentinel-node micrometastases (IBCSG 23-01): a phase 3 randomised controlled trial. Lancet Oncol 2013;14:297–305 23491275 10.1016/S1470-2045(13)70035-4 PMC3935346

[4] Giuliano AE, McCall L, Beitsch P, Whitworth PW, Blumencranz P, Leitch AM et al Locoregional recurrence after sentinel lymph node dissection with or without axillary dissection in patients with sentinel lymph node metastases: the American College of Surgeons Oncology Group Z0011 randomized trial. Ann Surg 2010;252:426–432 20739842 10.1097/SLA.0b013e3181f08f32 PMC5593421

[5] Giuliano AE, Ballman K, McCall L, Beitsch P, Whitworth PW, Blumencranz P et al Locoregional recurrence after sentinel lymph node dissection with or without axillary dissection in patients with sentinel lymph node metastases: long-term follow-up from the American College of Surgeons Oncology Group (Alliance) ACOSOG Z0011 randomized trial. Ann Surg 2016;264:413–420 27513155 10.1097/SLA.0000000000001863 PMC5070540

[6] Giuliano AE, Hunt KK, Ballman KV, Beitsch PD, Whitworth PW, Blumencranz PW et al Axillary dissection *versus* no axillary dissection in women with invasive breast cancer and sentinel node metastasis: a randomized clinical trial. JAMA 2011;305:569–575 21304082 10.1001/jama.2011.90 PMC5389857

[7] Giuliano AE, Ballman KV, McCall L, Beitsch PD, Brennan MB, Kelemen PR et al Effect of axillary dissection *versus* no axillary dissection on 10-year overall survival among women with invasive breast cancer and sentinel node metastasis: the ACOSOG Z0011 (Alliance) randomized clinical trial. JAMA 2017;318:918–926 28898379 10.1001/jama.2017.11470 PMC5672806

[8] Huang TW, Su CM, Tam KW. Axillary management in women with early breast cancer and limited sentinel node metastasis: a systematic review and metaanalysis of real-world evidence in the post-ACOSOG Z0011 era. Ann Surg Oncol 2021;28:920–929 32705512 10.1245/s10434-020-08923-7

[9] Heiranizadeh N, Rafiei Shahamabadi M, Dehghan HR, Jafari-Nedooshan J, Kargar S, Zare M et al Comparing early-stage breast cancer patients with sentinel lymph node metastasis with and without completion axillary lymph node dissection: a systematic review and meta-analysis. Asian Pac J Cancer Prev 2022;23:2561–2571 36037108 10.31557/APJCP.2022.23.8.2561 PMC9741898

[10] Mannu G, Beake G, Berry R, Dodwell D, Hills R, McGale P et al Abstract GS02-05 overview of axillary treatment in early breast cancer: patient-level meta-analysis of long- term outcomes among 20,273 women in 29 randomised trials. Cancer Res 2024;84(Suppl):GS02-05

[11] Glechner A, Wöckel A, Gartlehner G, Thaler K, Strobelberger M, Griebler U et al Sentinel lymph node dissection only versus complete axillary lymph node dissection in early invasive breast cancer: a systematic review and meta-analysis. Eur J Cancer 2013;49:812–825 23084155 10.1016/j.ejca.2012.09.010

[12] DiSipio T, Rye S, Newman B, Hayes S. Incidence of unilateral arm lymphoedema after breast cancer: a systematic review and meta-analysis. Lancet Oncol 2013;14:500–515 23540561 10.1016/S1470-2045(13)70076-7

[13] Wang L, Guyatt GH, Kennedy SA, Romerosa B, Kwon HY, Kaushal A et al Predictors of persistent pain after breast cancer surgery: a systematic review and meta-analysis of observational studies. Can Med Assoc J 2016;188:E352–E361 27402075 10.1503/cmaj.151276 PMC5047835

[14] Lee J, Jung JH, Kim WW, Kang B, Keum H, Chae YS et al Ten-year oncologic outcomes in T1-3N1 breast cancer after targeted axillary sampling: a retrospective study. Ann Surg Oncol 2023;30:4669–4677 36828929 10.1245/s10434-023-13191-2

[15] Bhattacharya I, Coles C, Doughty J, Irvine T, Makris A, Palmieri C et al Neoadjuvant chemotherapy: multidisciplinary guidance 2023. https://associationofbreastsurgery.org.uk/media/yuhlamsi/neaoadjuvant-chemotherapy-manual-v1.pdf (accessed 5 March 2023)

[16] National Comprehensive Cancer Network (NCCN) . NCCN guidelines version 4.2025: invasive breast cancer, considerations for surgical axillary staging. https://www.nccn.org/professionals/physician_gls/pdf/breast.pdf (accessed 1 December 2024)

[17] Boughey JC, Ballman KV, Le-Petross HT, McCall LM, Mittendorf EA, Ahrendt GM et al Identification and resection of clipped node decreases the false-negative rate of sentinel lymph node surgery in patients presenting with node-positive breast cancer (T0–T4, N1–N2) who receive neoadjuvant chemotherapy: results from ACOSOG Z1071 (Alliance). Ann Surg 2016;263:802–807 26649589 10.1097/SLA.0000000000001375 PMC4777661

[18] van Nijnatten TJA, Simons JM, Smidt ML, van der Pol CC, van Diest PJ, Jager A et al A novel less-invasive approach for axillary staging after neoadjuvant chemotherapy in patients with axillary node-positive breast cancer by combining radioactive iodine seed localization in the axilla with the sentinel node procedure (RISAS): a Dutch prospective multicenter validation study. Clin Breast Cancer 2017;17:399–402 28487053 10.1016/j.clbc.2017.04.006

[19] Donker M, Straver ME, Wesseling J, Loo CE, Schot M, Drukker CA et al Marking axillary lymph nodes with radioactive iodine seeds for axillary staging after neoadjuvant systemic treatment in breast cancer patients: the MARI procedure. Ann Surg 2015;261:378–382 24743607 10.1097/SLA.0000000000000558

[20] Caudle AS, Yang WT, Krishnamurthy S, Mittendorf EA, Black DM, Gilcrease MZ et al Improved axillary evaluation following neoadjuvant therapy for patients with node-positive breast cancer using selective evaluation of clipped nodes: implementation of targeted axillary dissection. J Clin Oncol 2016;34:1072–1078 26811528 10.1200/JCO.2015.64.0094 PMC4933133

[21] Fairhurst K, McIntosh SA, Cutress RI, Potter S; Our Citable Collaborators. Current axillary management of patients with early breast cancer and low-volume nodal disease undergoing primary surgery: results of a United Kingdom national practice survey. Breast Cancer Res Treat 2024;206:465–471 38724821 10.1007/s10549-024-07328-4 PMC11208217

[22] Mullan R, McIntosh S, Potter S. Surgical techniques should be developed together: exploring the introduction of targeted axillary dissection. Ann Surg Oncol 2025;32:311–952. doi:10.1245/s10434-025-17646-6. The American Society of Breast Surgeons Official Proceedings, Volume XXVI 2025 Annual Meeting Scientific Session Abstracts. Supp 2 40627106

[23] Butterworth JW, Boshier PR, Mavroveli S, Van Lanschot JB, Sasako M, Reynolds JV et al Challenges to quality assurance of surgical interventions in clinical oncology trials: a systematic review. Eur J Surg Oncol 2021;47:748–756 33059943 10.1016/j.ejso.2020.10.002

[24] Blencowe NS, Mills N, Cook JA, Donovan JL, Rogers CA, Whiting P et al Standardizing and monitoring the delivery of surgical interventions in randomized clinical trials. Br J Surg 2016;103:1377–1384 27462835 10.1002/bjs.10254 PMC5132147

[25] Potter S, Mullan R, Cain H, John ES, Barry P, Masannat Y et al A consensus process to agree surgical quality assurance processes within a pragmatic, multicentre randomised clinical trial comparing targeted axillary dissection and axillary node clearance: the TADPOLE-TOGETHER project. BMJ Open 2025;15:e095774 10.1136/bmjopen-2024-095774 PMC12142125 40461151

[26] Hsieh HF, Shannon SE. Three approaches to qualitative content analysis. Qual Health Res 2005;15:1277–1288 16204405 10.1177/1049732305276687

[27] Guyatt GH, Oxman AD, Kunz R, Atkins D, Brozek J, Vist G et al GRADE guidelines: 2. Framing the question and deciding on important outcomes. J Clin Epidemiol 2011;64:395–400 21194891 10.1016/j.jclinepi.2010.09.012

[28] Harris PA, Taylor R, Thielke R, Payne J, Gonzalez N, Conde JG. Research Electronic Data Capture (REDCap)—a metadata-driven methodology and workflow process for providing translational research informatics support. J Biomed Inform 2009;42:377–381 18929686 10.1016/j.jbi.2008.08.010 PMC2700030

[29] Brookes ST, Macefield RC, Williamson PR, McNair AG, Potter S, Blencowe NS et al Three nested randomized controlled trials of peer-only or multiple stakeholder group feedback within Delphi surveys during core outcome and information set development. Trials 2016;17:409 27534622 10.1186/s13063-016-1479-x PMC4989325

[30] Potter S, Brookes ST, Holcombe C, Ward JA, Blazeby JM. Exploring methods the for selection and integration of stakeholder views in the development of core outcome sets: a case study in reconstructive breast surgery. Trials 2016;17:463 27664072 10.1186/s13063-016-1591-y PMC5034558

[31] Williamson PR, Altman DG, Bagley H, Barnes KL, Blazeby JM, Brookes ST et al The COMET handbook: version 1.0. Trials 2017;18:280 28681707 10.1186/s13063-017-1978-4 PMC5499094

[32] Avery K, Blazeby J, Wilson N, Macefield R, Cousins S, Main B et al Development of reporting guidance and core outcome sets for seamless, standardised evaluation of innovative surgical procedures and devices: a study protocol for content generation and a Delphi consensus process (COHESIVE study). BMJ Open 2019;9:e029574 10.1136/bmjopen-2019-029574 PMC6747635 31515426

[33] Potter S, Holcombe C, Ward JA, Blazeby JM; BRAVO Steering Group. Development of a core outcome set for research and audit studies in reconstructive breast surgery. Br J Surg 2015;102:1360–1371 26179938 10.1002/bjs.9883 PMC5034747

[34] Potter S, Avery K, Ahmed R, de Boniface J, Chatterjee S, Dodwell D et al Protocol for the development of a core outcome set and reporting guidelines for locoregional treatment in neoadjuvant systemic breast cancer treatment trials: the PRECEDENT project. BMJ Open 2024;14:e084488 10.1136/bmjopen-2024-084488 PMC11033665 38643011

[35] Boutron I, Moher D, Altman DG, Schulz KF, Ravaud P; CONSORT Group. Extending the CONSORT statement to randomized trials of nonpharmacologic treatment: explanation and elaboration. Ann Intern Med 2008;148:295–309 18283207 10.7326/0003-4819-148-4-200802190-00008

[36] Alkner S, Wieslander E, Lundstedt D, Berg M, Kristensen I, Andersson Y et al Quality assessment of radiotherapy in the prospective randomized SENOMAC trial. Radiother Oncol 2024;197:110372 38866204 10.1016/j.radonc.2024.110372

[37] Goyal A, Mann GB, Fallowfield L, Duley L, Reed M, Dodwell D et al POSNOC—POsitive Sentinel NOde: adjuvant therapy alone *versus* adjuvant therapy plus clearance or axillary radiotherapy: a randomised controlled trial of axillary treatment in women with early-stage breast cancer who have metastases in one or two sentinel nodes. BMJ Open 2021;11:e054365 10.1136/bmjopen-2021-054365 PMC8640630 34857578

[38] Goyal A, Nicholls S, Marshall A, Hammonds N, Wheatley D, Elsberger B et al Abstract OT1-09-02: ATNEC: a multicentre, randomized trial investigating whether axillary treatment can be avoided in T1-3N1M0 breast cancer patients with no residual cancer in the axillary lymph nodes after neoadjuvant chemotherapy. Cancer Res 2023;83(Suppl):OT1-09-02

[39] Blencowe NS, Skilton A, Gaunt D, Brierley R, Hollowood A, Dwerryhouse S et al Protocol for developing quality assurance measures to use in surgical trials: an example from the ROMIO study. BMJ Open 2019;9:e026209 10.1136/bmjopen-2018-026209 PMC6429900 30826769

[40] Harris A, Butterworth JB, Boshier PR, Mavroveli S, Vadhwana B, Peters CJ et al Development of a reliable surgical quality assurance tool for gastrectomy in oncological trials. Gastric Cancer 2024;27:876–883 38761290 10.1007/s10120-024-01503-8 PMC11193692

[41] McIntosh S, Dubsky PC, Avery K, de Boniface J, Dodwell D, Finestone S et al Setting a precedent: development of a core outcome set for locoregional treatment outcome reporting in neoadjuvant breast cancer trials. J Clin Oncol 2025;43:e12570–e12570

