# Supplementary material for: Safe and transparent introduction and evaluation of targeted axillary dissection in patients with node-positive breast cancer undergoing primary surgery: international consensus process
Source: BJS Open. 2025 Oct 30;9(6):zraf121. doi: 10.1093/bjsopen/zraf121 (PMC12574671; doi:10.1093/bjsopen/zraf121)
Supplement: zraf121_Supplementary_Data [file zraf121_supplementary_data.zip › Supplementary_Material.docx]

**Title: An international consensus process to support the safe and transparent introduction and evaluation of targeted axillary dissection in patients with node positive breast cancer having primary surgery**

Authors: Shelley Potter^1,2^, Ruth Mullan^3^, Henry Cain^4^, Edward R St John^5,6^, Peter Barry^7^, Yazan Massanat^8^, James Harvey^9^, Katherine Fairhurst^1,4^, Adrienne Morgan^10^, Margaret Perkins^10^, Gregory Bruce Mann^11-14^, Jocelyn Lippey^14-16^, Katherine Cowan^17^, Natalie Blencowe^18^*, Stuart A McIntosh^3^*on behalf of the TADPOLE Trial Management Group^§^ and the TADPOLE-TOGETHER collaborative^§^

^1^Bristol Surgical and Perioperative Care Complex Intervention Collaboration, Translational Health Sciences, Bristol Medical School. Learning and Research Building, Southmead Hospital, Bristol, BS10 5NB

^2^Bristol Breast Care Centre, North Bristol NHS Trust, Southmead Road, Bristol, BS10 5NB, UK

^3^Patrick G Johnston Centre for Cancer Research, Queen’s University Belfast, 97 Lisburn Road, Belfast, BT9 7AE

^4^The Newcastle upon Tyne Hospitals NHS Foundation Trust, Newcastle, UK, ^5^Portsmouth Hospitals University NHS Trust, Portsmouth, UK

^6^Research Group in Breast Health, University of Portsmouth, Portsmouth, UK,

^7^The Royal Marsden NHS Foundation Trust, London, UK

^8^Broomfield Hospital, Mid and South Essex NHS Trust

^9^Manchester University Hospitals NHS Foundation Trust, Manchester UK

^10^Independent Cancer Patients’ Voice, London, UK

^11^The Royal Melbourne Hospital, Parkville, VIC, Australia

^12^The Royal Women's Hospital, Parkville, VIC, Australia

^13^Breast Cancer Trials, Newcastle, NSW, Australia

^14^Department of Surgery, The University of Melbourne, Parkville, VIC, Australia

^15^St Vincent's BreastScreen, St Vincent's Hospital Melbourne, Melbourne, VIC, Australia. Department of Surgery

^16^St Vincent's Hospital Melbourne, Melbourne, VIC, Australia,

^17^Katherine Cowan Consulting. Southend on Sea, UK

^18^Centre for Surgical Research, Population Health Sciences, Bristol Medical School, Canynge Hall, Bristol BS8 2PS

**Corresponding author.** Shelley Potter, Translational Health Sciences, Bristol Medical School. Learning and Research Building, Southmead Hospital, Bristol, BS10 5NB

**ORCID ID 0000-0002-6977-312X**; **Twitter** @drshelleypotter

**Supplementary Materials - Index**

| **Supplementary Figures and Tables** |  |
| --- | --- |
| **Supplementary Table 1**: Consensus achieved after the Round 2 Delphi Survey regarding the component steps and standardisation of a primary TAD procedure | *Page 3* |
| **Supplementary Table 2**: Consensus achieved after the Round 2 Delphi Survey regarding mandatory and prohibited steps of an axillary clearance | *page 5* |
| **Supplementary Table 3:** TADPOLE Surgical Case Report Form Items | *page 6* |
| **Supplementary Table 4:** Consensus achieved after the Round 2 Delphi survey regarding the management of difficult situations that may occur when performing a primary TAD procedure | *page 7* |
| **Supplementary Table 5**: Consensus achieved following the Round 2 Delphi survey regarding surgeon experience and training to participate in the TADPOLE trial | *page 8* |
| **Supplementary Appendixes** |  |
| TADPOLE TOGETHER Round 1 Survey | *page 9* |
|  |  |
|  |  |

**Supplementary Table 1: Consensus achieved after the Round 2 Delphi Survey regarding the component steps and standardisation of a primary TAD procedure**

| **COMPONENT STEP OF PRIMARY TAD** | | | **STANDARDISATION OF COMPONENT STEP** | | |
| --- | --- | --- | --- | --- | --- |
| **COMPONENT STEP** | **RESULT AFTER ROUND 2 DELPHI** | **OUTCOME** | **DETAILS OF HOW COMPONENT STEP IS PERFORMED** | **RESULT AFTER ROUND 2 DELPHI** | **DECISION REGARDING STANDARDISATION OF COMPONENT STEP** |
| **The involved lymph node is localised prior to surgery** | CONSENSUS  136/140 (97%) | **MANDATORY** | **METHOD** for localising the node | CONSENSUS  107/136 (79%) | As per local unit practice |
|  |  |  | **TIMING** of localisation | CONSENSUS  108/136 (79%) | As per local unit practice |
| **There is confirmation that the involved node is correctly localised prior to surgery** | CONSENSUS 97/139  (70%) | **MANDATORY** | **METHOD** for confirming that the INVOLVED NODE has been CORRECTLY LOCALISED | CONSENSUS  105/136 (77%) | As per local unit practice |
|  |  |  | **TIMING** of confirmation that THE INVOLVED NODE has been CORRECTLY LOCALISED | CONSENSUS  100/136 (74%) | As per local unit practice |
| **The skin is marked to demonstrate the position of the localised node prior to surgery** | CONSENSUS  123/140 (88%) | **OPTIONAL** | **HOW** the SKIN is MARKED | OPTIONAL STEP | As per local unit practice |
|  |  |  | **WHEN** the SKIN is MARKED | OPTIONAL STEP | As per local unit practice |
| **A sentinel node biopsy is performed as part of the primary TAD procedure** | CONSENSUS  135/140 (96%) | **MANDATORY** | **TECHNIQUE** used for sentinel node localisation | **NO CONSENSUS**  As per local unit practice  Dual tracer technique (blue dye and radioactive isotope)  Single tracer (radioactive isotope)  Single tracer (any) | 80/131 (61%)  48/131 (37%)  3/131 (2%)  0 (0%) |
|  |  |  | **LYMPHOSCINTOGRAPHY** is performed | CONSENSUS  93/131 (71%) | As per local unit practice  (Not essential) |
|  |  |  | **POSITION** of the sentinel node localisation INJECTION in the breast | CONSENSUS  100/131 (76%) | As per local unit practice |
|  |  |  | SNB is performed **BEFORE** removal of the LOCALISED node | CONSENSUS  102/131 (78%) | As per local surgeon practice (Not essential) |
| **The localised node is removed during the TAD procedure** | CONSENSUS  138/140 (99%) | **MANDATORY** | **METHOD** for intra-operative confirmation of removal of the localised involved lymph node | **NO CONSENSUS**  Not essential (1-3)  Equivocal (4-6)  Essential (7-9) | 16%  16%  68% |
| **Removal of the localised node is confirmed intra-operatively** | CONSENSUS  127/139 (91%) | **MANDATORY** |  |  |  |
| **All sentinel nodes are removed during the primary TAD procedure** | CONSENSUS  130/139 (94%) | **MANDATORY** | Not applicable | All sentinel nodes are removed during the primary TAD procedure | |
| **Any palpably ABNORMAL lymph nodes are removed during the primary TAD procedure** | CONSENSUS  99/139 (71%) | **MANDATORY** | Not applicable | Any palpably ABNORMAL lymph nodes are removed during the primary TAD procedure | |
| **A specified MINIMUM number of nodes are removed during primary TAD procedure** | CONSENSUS  103/140 (74%) | **NON-MANDATORY** | Not applicable | No specified minimum number of nodes to be removed during a primary TAD procedure | |
| **The TOTAL NUMBER of lymph nodes that can be removed in the TAD group is limited** | CONSENSUS  101/140 (72%) | **NON-MANDATORY** | Not applicable | No specified maximum number of nodes that can be removed during a primary TAD procedure | |
| **Additional NORMAL non-sentinel lymph nodes (not including localised and/or sentinel nodes) are removed** | CONSENSUS  114/140 (81%) | **PROHIBITED** | Not applicable | No additional normal non-sentinel lymph nodes should be removed during a primary TAD procedure | |
| **A drain is inserted during the procedure** | CONSENSUS 107/140 (76%) | **OPTIONAL** | Not applicable | OPTIONAL STEP | As per local unit practice |
| **Local anaesthetic is used during the procedure** | CONSENSUS 110/140 (78%) | **OPTIONAL** | Not applicable | OPTIONAL STEP | As per local unit practice |

**Supplementary Table 2: Consensus achieved after the Round 2 Delphi Survey regarding mandatory and prohibited steps of an axillary clearance**

| **COMPONENT STEP** | **CONSENSUS** | **MANDATORY, OPTIONAL OR PROHIBITED** |
| --- | --- | --- |
| The axillary vein and other important structures are seen and preserved during the procedure | CONSENSUS  129/131 (98%) | **MANDATORY** |
| A level 1 and 2 clearance is performed | CONSENSUS  122/131 (93%) | **MANDATORY** |
| A level 3 clearance is routinely performed in the ABSENCE OF PALPABLE DISEASE | CONSENSUS  93/131 (71%) | **PROHIBITED** |
| Clips are placed at the upper boundary of the dissection to guide radiotherapy | CONSENSUS  97/131 (74%) | **OPTIONAL** |
| A drain is used | CONSENSUS  106/131 (81%) | **OPTIONAL** |

**Supplementary Table 3: TADPOLE Surgical Case Report Form Items**

|  | **OUTCOME FOLLOWING ROUND 2 OF DELPHI SURVEY** | | **OUTCOME FOLLOWING CONSENSUS MEETING**  **CRF REPORTING** |
| --- | --- | --- | --- |
| **DATA ITEM** | **CONSENSUS ACHIEVED** | **ESSENTIAL TO REPORT IN FUTURE TRIAL** |  |
| The number of involved lymph nodes biopsied and clipped (TAD group) | CONSENSUS | ESSENTIAL (125/130, 96%) | ESSENTIAL |
| The method of sentinel node localisation used (TAD group) | CONSENSUS | ESSENTIAL (126/130, 97%) | ESSENTIAL |
| The method for localising the involved node in the TAD group (e.g. wire, SaviScout etc) | CONSENSUS | ESSENTIAL (121/130, 93%) | ESSENTIAL |
| The number of involved lymph nodes localised | CONSENSUS | ESSENTIAL (127/130, 98%) | ESSENTIAL |
| The timing of localisation | NO CONSENSUS | Not essential (1-3) 23/130 (18%)  Equivocal (4-6) 67/130 (52%)  Essential (7-9) 40/130 (31%) | ESSENTIAL TO REPORT if node marked at time of biopsy (one stage) or after biopsy results available (two stage) localisation |
| The total number of lymph nodes removed in the TAD group | CONSENSUS | ESSENTIAL (128/130, 98%) | ESSENTIAL |
| Whether the localisation device/clip is identified and removed (TAD group) | CONSENSUS | ESSENTIAL (128/130, 98%) | ESSENTIAL |
| Whether the localised node was the sentinel node intraoperatively in the TAD group | CONSENSUS | ESSENTIAL (123/130, 95%) | ESSENTIAL |
| The method for confirming removal of the localised node (TAD group) | CONSENSUS | ESSENTIAL (116/130, 89%) | ESSENTIAL |
| The duration of the axillary procedure (both groups) | NO CONSENSUS | Not essential (1-3) 37/130 (28%)  Equivocal (4-6) 65/130 (50%)  Essential (7-9) 28/130 (22%) | NOT ESSENTIAL TO REPORT |

**Supplementary Table 4: Consensus achieved after the Round 2 Delphi survey regarding the management of difficult situations that may occur when performing a primary TAD procedure**

| **Situation** | **Delphi Outcome** | **Consensus-based management strategy** | |
| --- | --- | --- | --- |
| If the sentinel node localisation technique FAILS (e.g. no blue dye/isotope in the axilla) in a patient having a PRIMARY TAD | CONSENSUS 114/131 (87%) | Remove the localised involved node and perform a 4-node sample (including any palpably abnormal nodes) | |
| If the clip/localisation device is identified, but it is NOT in the involved node in a patient having a PRIMARY TAD | NO CONSENSUS | Perform the sentinel node biopsy and remove any palpably abnormal nodes and nothing else  Perform the SNB, remove any palpably abnormal nodes AND any nodes close to the clip/localisation device  Perform an axillary node clearance | 32/131 (24%)  83/131 (63%)  16 (12%) |
| If the clip/localisation device CANNOT BE IDENTIFIED intraoperatively in a patient having a primary TAD | CONSENSUS  105/131 (80%) | Perform a SNB and remove any palpably abnormal nodes and await histology results (further surgery likely to be needed if no involved nodes are identified on pathology) | |
| If NEITHER the SENTINEL NODE(S) NOR the LOCALISED NODE can be identified intraoperatively | NO CONSENSUS | Perform a 4 node sample including removal of any palpably abnormal nodes  Perform an axillary node clearance | 77/131 (59%)  54/131 (41%) |
| Intra-operative findings are highly suspicious for UNANTICIPATED extensive disease in a patient randomised to PRIMARY TAD | NO CONSENSUS | Remove localised node, sentinel node and any palpably abnormal nodes and await histology (patient may require further surgery)  Perform an axillary node clearance | 81/131 (62%)  50/131 (38%) |

**Supplementary Table 5: Consensus achieved following the Round 2 Delphi survey regarding surgeon experience and training to participate in the TADPOLE trial**

| **RECOMMENDED TRAINING AND/OR EXPERIENCE** | **CONSENSUS AFTER ROUND 2 DELPHI SURVEY** | **DELPHI OUTCOME** |
| --- | --- | --- |
| Watched TADPOLE training videos and/or attended TADPOLE webinar | CONSENSUS  108/130 (83%) | ESSENTIAL |
| Were familiar with performing TAD in patients following neoadjuvant treatment and had performed a pre-specified number of cases | No consensus  Not essential (1-3)  Equivocal (4-6)  Essential (7-9) | 10/130 (8%)  31/130 (24%)  89/130 (68%) |
| Are participating in the ATNEC study | CONSENSUS  93/130 (72%) | NOT ESSENTIAL |
| How many TAD procedures do you think a surgeon should have performed before they can participate in the TADPOLE trial? | No consensus  No pre-specified minimum number of TAD procedures  At least 5  6 - 10  >10 | 75/130 (58%)  31/130 (24%)  8/130 (6%)  16/130 (12%) |
