# Supplementary material for: Safe and transparent introduction and evaluation of targeted axillary dissection in patients with node-positive breast cancer undergoing primary surgery: international consensus process
Source: BJS Open. 2025 Oct 30;9(6):zraf121. doi: 10.1093/bjsopen/zraf121 (PMC12574671; doi:10.1093/bjsopen/zraf121)
Supplement: zraf121_Supplementary_Data [file zraf121_supplementary_data.zip › TADPOLETOGETHERSurvey.pdf]

TADPOLE TOGETHER Round 1 Survey

TADPOLE-TOGETHER

Help us agree how axillary surgery should be standardised in the UK TADPOLE trial!

Do we still need to perform an axillary node clearance in patients with low volume nodal disease having primary surgery?

In ~20% of patients, their breast cancer will have spread to the axillary nodes at diagnosis. Currently, NICE recommend that if these patients have having surgery as their initial (primary) treatment, they should have an axillary node clearance (ANC) even if they have limited nodal disease (defined as 1-2 involved nodes detected on imaging).

One in three patients, however, will experience major life changing complications after an ANC. These include permanent swelling of the arm (lymphoedema) and long-term pain. These complications dramatically affect patients' quality of life and as they are life-long, are costly to the NHS.

Such extensive surgery, however, may not be necessary as the recent Early Breast Cancer Trialists' Collaborative Group axillary treatment meta-analysis has shown extent of surgery has NO IMPACT on breast cancer survival.

A targeted axillary dissection (TAD) may be a better option for many patients as it may reduce the risk of life-long complications without impacting oncological outcomes.

What is TADPOLE?

TADPOLE is an NIHR-HTA funded multicentre, pragmatic, randomised clinical trial that aims to determine if TAD is a safe alternative to ANC in breast cancer patients with low volume nodal disease having primary surgery. Participants will be randomised 2:1 into TAD and ANC groups. The co-primary endpoints are i) patient-reported and objective lymphoedema at 12 months and ii) locoregional recurrence at 5 years. The trial will aim to recruit 861 participants from 40 UK sites and is planned to open Early 2025.

What is TADPOLE-TOGETHER?

TADPOLE is a trial comparing two types of axillary surgery. It is therefore vital to ensure that both types of axillary surgery (TAD and ANC) will be performed in a sufficiently standardised way within the trial so that the results will be accepted and implemented by the surgical community. This is particularly important for TAD as our national practice survey has highlighted variation.

We need you to help us agree how to perform TAD and ANC in the TADPOLE Trial.

We want TADPOLE to reflect surgical practice and for the surgical community to help us agree how TAD and ANC should be performed within the TADPOLE trial.

What would I need to do?

We would like you to complete two rounds of an online Delphi survey to help us determine which components of the surgical procedure need to be standardised in TADPOLE and which elements can be flexible and reflect local practice or surgeon preference. The survey will take no more than 15-20 minutes to complete.

We will analyse the results of the first survey and you will then be asked to complete the survey again. This survey will contain feedback on how important other breast surgeons felt it was to standardise each part of the operation together with your own scores. You will be asked to re-score again based on the feedback received. It is important to complete both surveys to help us reach agreement about which parts should be standardised in the Trial.

All surgeons who complete BOTH surveys will be offered PUBMED citable collaborator status on the future publication and will be invited to attend an in-person workshop at which the final approach to axillary surgery in the TADPOLE trial is agreed.

Who should take part?

Consultant surgeons and senior trainees routinely performing axillary surgery as part of breast cancer treatment. A senior trainee is defined as someone who has received their CCT or is predicted to receive it within the next 12 months.

Survey responses will be anonymous. We will collect your e-mail address, but this is just to allow us to send you the 2nd round questionnaire, it will not be used for any other reason.

Data management

All data will be stored securely on REDCap hosted by the University of Bristol. Only members of the TADPOLE-TOGETHER study team will have access to the data.

E-mail addresses will be retained until the TADPOLE-TOGETHER study is published so we can ensure correct details are provided for PUBMED citable collaborator status. This will be no later than 18 months following completion of the study. All e-mail addresses will then be deleted.

A fully anonymised dataset will be retained for a maximum of five years.

How do I get involved?

Please complete the Round 1 survey below.

Taking part is voluntary. If you decide that you no longer wish to participate in the study, please contact the Chief Investigator by e-mail (shelley,potter@bristol.ac.uk). It will be possible to withdraw your survey data until the Round 1 analysis is performed (planned October 2024).

Complaints

This study has been reviewed and given a favourable opinion by the University of Bristol Faculty of Health Sciences Research Ethics Committee (reference: 20773) If you have a complaint about any aspect of this study, please contact the University of Bristol Research Governance Team (research-governance@bristol.ac.uk).

Questions?

If you have any questions about the study, please contact the Chief Investigator of TADPOLE, Professor Shelley Potter (shelley.potter@bristol.ac.uk).

Many thanks for your help with this important study

Shelley Potter

TADPOLE Chief Investigator on behalf of the TADPOLE-TOGETHER team.

ABOUT YOU

We would like to collect some basic information about you and your experience with different surgical techniques.

We also ask that you provide an e-mail address so that we can send you the 2nd survey. We will not use your e-mail address for any other purpose.

Are you

- ◯ A Consultant/Attending Surgeon or equivalent
- ◯ A senior surgical trainee (within 1 year of obtaining Certificate of Completion of Training (CCT) or post CCT in the UK) or undertaking a specialist breast surgery fellowship
- ◯ An Associate Specialist/Senior Speciality Doctor with independent practice
- ◯ Other role

Do you routinely treat patients with breast cancer including performing axillary surgery? ◯ Yes ◯ No

How many years experience do you have as a consultant/attending surgeon?

◯ < 5
◯ 5-10
◯ 11-15
◯ 15-20
◯ >20
◯ Still in training

Geographical region of practice

◯ UK
◯ Europe
◯ North America
◯ Australia or New Zealand
◯ Africa
◯ Asia
◯ Other

What region in the UK do you work in?

◯ Scotland
◯ North West
◯ North East
◯ Yorkshire
◯ Midlands
◯ London
◯ South West
◯ South East
◯ Wales
◯ Northern Ireland

Please provide your e-mail address so that we can send you the Round 2 Survey.
We will not use your e-mail address for any other purpose ______________________________

YOUR EXPERIENCE OF TARGETED AXILLARY DISSECTION

Are you performing targeted axillary dissection (TAD) in your routine clinical practice (please tick all that apply)?

☐ Yes - in the neoadjuvant setting
☐ Yes - in ALL patients with low volume nodal disease (cN0, 1-2 involved node on USS) having primary surgery
☐ Yes, in SELECTED patients with low volume nodal disease having primary surgery
☐ Not performing TAD in my practice currently

Approximately how many TAD procedures have you performed to date?

◯ None ◯ < 10 ◯ 10-20 ◯ 21-50 ◯ >50

Is your Unit currently participating in the UK ATNEC study?

◯ Yes ◯ No ◯ In set up

HOW SHOULD A PRIMARY TAD BE PERFORMED?

The TADPOLE trial aims to compare targeted axillary dissection (TAD) and axillary node clearance (ANC) in patients with node positive breast cancer having primary surgery.

We want to ensure that everyone is performing the primary TAD procedure in a consistent way across all the sites participating in the TADPOLE trial.

Below is a list of the component steps of a TAD procedure identified from the literature and expert opinion.

For each step, please indicate whether you feel that it should be:

i) MANDATORY - i.e. MUST be performed in ALL cases of TAD in the TADPOLE trial
ii) OPTIONAL - i.e. up to individual surgeons whether this step should be done/not done as part of the procedure
iii) PROHIBITED - i.e. MUST NOT be performed in TAD patients

If you tick that a step is MANDATORY you will be asked to answer some questions about how it should be delivered during the trial (ie. ranging from completely standardised to completely flexible).

Please bear in mind TADPOLE is a pragmatic multicentre trial so any recommendations for axillary surgery need to applicable to ALL 40+ SITES participating in the trial.

TERMINOLOGY
In this survey
MARKING of the node refers to insertion of a radiology clip (e.g. at time of biopsy) that then needs a further procedure to identify the involved node prior to surgery.

LOCALISATION of the node refers to identifying the involved node in preparation for removal in theatre. This may involve a wire, tattoo or localisation device (e.g. Magseed, SaviScout or Pintuition)

	Mandatory	Optional	Prohibited
The involved lymph node is localised prior to surgery	○	○	○
There is confirmation that the involved node is correctly localised prior to surgery	○	○	○

The skin is marked to demonstrate the position of the localised node prior to surgery	◯	◯	◯
A sentinel node biopsy is performed as part of the primary TAD procedure	◯	◯	◯
The localised node is removed during the TAD procedure	◯	◯	◯
Removal of the localised node is confirmed intra-operatively	◯	◯	◯
All sentinel nodes are removed during the primary TAD procedure	◯	◯	◯
Any palpably ABNORMAL lymph nodes are removed during the primary TAD procedure	◯	◯	◯
A specified MINIMUM number of nodes are removed during primary TAD procedure	◯	◯	◯
The TOTAL NUMBER of lymph nodes that can be removed in the TAD group is limited	◯	◯	◯
Additional NORMAL non-sentinel lymph nodes (not including localised and/or sentinel nodes) are removed	◯	◯	◯
A drain is inserted during the procedure	◯	◯	◯
Local anaesthetic is used during the procedure	◯	◯	◯

Are there ANY OTHER STEPS that you think should be MANDATORY when performing a PRIMARY TAD (i.e. a TAD in patients having primary surgery)?

◯ Yes ◯ No

Please give details of any additional MANDATORY steps that should be performed as part of a PRIMARY TAD procedure

Are there ANY OTHER STEPS that you think should be PROHIBITED when performing a PRIMARY TAD (i.e. a TAD in patients having primary surgery)?

◯ Yes ◯ No

Please give details of any additional steps that should be PROHIBITED when performing a PRIMARY TAD procedure

If LOCALISING THE INVOLVED NODE PRIOR TO SURGERY is MANDATORY. How essential is it that the following factors are standardised (i.e. done in a specific way) vs as per local unit practice:

1 = NOT essential
9 = ABSOLUTELY essential

	1 Not essential	2	3	4	5	6	7	8	9 Absolutely essential
The METHOD for localising the node (e.g. specifying a specific method (e.g wire) vs local unit practice)	◯	◯	◯	◯	◯	◯	◯	◯	◯
The TIMING of localisation (e.g at a specific time point vs standard unit practice)	◯	◯	◯	◯	◯	◯	◯	◯	◯

What METHOD FOR LOCALISING THE INVOLVED NODE should be mandated in the trial?

◯ Clip and wire localisation
◯ Localisation device (MagSeed, SaviScout, Pintuition, iodine seed etc)
◯ Carbon tattooing
◯ Other (please give details)

If other method, please give details

____

If it is necessary to standardise the TIMING of the LOCALISATION of the involved node, when should this be?

◯ At the time of biopsy (before nodal involvement confirmed)
◯ At any time after nodal involvement is confirmed
◯ On the day of surgery
◯ Other (please give details)

Other standard timing of localisation of the involved node prior to surgery

____

If CONFIRMATION THAT THE INVOLVED NODE HAS BEEN CORRECTLY LOCALISED PRIOR TO SURGERY is MANDATORY, how essential is it that the following factors are are standardised (i.e. done in a specific way) vs as per local unit practice:

1 = NOT essential
9 = ABSOLUTELY essential

	1 Not essential	2	3	4	5	6	7	8	9 Absolutely essential
HOW the confirmation is performed (e.g USS vs detection of signal from tag)	◯	◯	◯	◯	◯	◯	◯	◯	◯
WHEN the confirmation is performed	◯	◯	◯	◯	◯	◯	◯	◯	◯

HOW should confirmation of localisation be performed?

◯ Using USS following placement of wire/localisation device
◯ By detecting a signal if localisation device is used
◯ Other method (please specify)

Other method of confirming localisation

____

WHEN should confirmation of localisation be performed?

◯ At the point of wire/localisation device insertion
◯ On the day of surgery before patient is anaesthetised
◯ Both time points
◯ Other (please specify)

Other timepoints(s) when confirmation of localisation should be performed

____

If marking the skin to demonstrate the position of the localised node prior to surgery is MANDATORY, how essential is it that the following factors are are standardised (i.e. done in a specific way) vs as per local unit practice within the trial:

1 = NOT essential
9 = ABSOLUTELY essential

	1 Not essential	2	3	4	5	6	7	8	9 Absolutely essential
How the skin marking is performed (e.g USS vs detection of signal from tag)	○	○	○	○	○	○	○	○	○
When the skin marking is performed	○	○	○	○	○	○	○	○	○

Performing a SENTINEL NODE BIOPSY is a MANDATORY step in a primary TAD procedure

If performing a sentinel node biopsy as part of the primary TAD procedure is MANDATORY, how essential is it that the sentinel node localisation is performed in a standardised way rather than as per local Unit policy in the trial?

◯ 1 Not essential ◯ 2 ◯ 3 ◯ 4 ◯ 5 ◯ 6 ◯ 7 ◯ 8 ◯ 9 Absolutely essential

If it is absolutely essential that the sentinel node localisation is performed in a standardised way, how should it be performed?

◯ Dual tracer technique (blue dye and radioactive isotope) ◯ Other (please give details)

Please give details

____

If isotope is used as part of the sentinel node localisation, how essential is it that lymphoscintography is performed?

◯ 1 Not essential ◯ 2 ◯ 3 ◯ 4 ◯ 5 ◯ 6 ◯ 7 ◯ 8 ◯ 9 Absolutely essential

If performing a sentinel node biopsy as part of the primary TAD procedure is MANDATORY, how essential is it that the POSITION of the sentinel node localisation INJECTION in the breast is standardised in the trial?

◯ 1 Not essential ◯ 2 ◯ 3 ◯ 4 ◯ 5 ◯ 6 ◯ 7 ◯ 8 ◯ 9 Absolutely essential

Where should the localisation injection be?

◯ In the same quadrant as the cancer ◯ At the 12 o'clock position ◯ Other (please specify)

Please specific other standardisation

____

How essential is it that the SNB is performed BEFORE removal of the LOCALISED node?

◯ 1 Not essential ◯ 2 ◯ 3 ◯ 4 ◯ 5 ◯ 6 ◯ 7 ◯ 8 ◯ 9 Absolutely essential

Numbers of lymph nodes removed in a PRIMARY TAD and confirmation of removal of the localised node

If intra-operative confirmation of removal of the localised involved lymph node is MANDATORY, how essential is it that confirmation is done in a standardised way within the trial (e.g intraoperative radiograph vs detection of signal from in removed node)?

○ 1 Not essential ○ 2 ○ 3 ○ 4 ○ 5 ○ 6 ○ 7 ○ 8 ○ 9 Absolutely essential

---

If removal of a specified MINIMUM number of nodes during a primary TAD procedure is MANDATORY, how many lymph nodes should this be?

○ 1 ○ 2 ○ 3 ○ 4 ○ Other (please specify)

Other minimum number of lymph nodes to be removed

____

If TOTAL NUMBER of lymph nodes that can be removed in patients having PRIMARY TAD is limited, what should be the MAXIMUM number of nodes that can be removed?

○ 3 ○ 4 ○ 5 ○ 6 ○ Other (please specify)

Other maximum number of lymph nodes that could be removed

____

Do you have any other comments about how PRIMARY TAD should be performed?

MANAGEMENT OF DIFFICULT SITUATIONS IN PATIENTS HAVING PRIMARY TAD

We would like to establish consensus about how best to manage potential problems that may be encountered when performing a PRIMARY TAD.

Please consider the scenarios below and select what option you feel would be best in each one.

If the sentinel node localisation technique FAILS (e.g. no blue dye/isotope in the axilla) in a patient having a PRIMARY TAD, what would you consider the best course of action intraoperatively?

◯ Remove the localised involved node and perform a 4 node sample (including any palpably abnormal nodes)
◯ Perform an axillary node clearance
◯ Other (please give details below)

Details of intraoperative management of patient with a failed SNB localisation having PRIMARY TAD

If the clip/localisation device is identified, but it is NOT in the involved node in a patient having a PRIMARY TAD, what should be done intraoperatively?

◯ Perform the sentinel node biopsy and remove any palpably abnormal nodes and nothing else
◯ Perform the SNB, remove any palpably abnormal nodes AND any nodes close to the clip/localisation device
◯ Perform an axillary node clearance
◯ Other (please give details below)

Details of intraoperative management of patient having PRIMARY TAD in whom clip/localisation device is NOT in the node

If the clip/localisation device CANNOT BE IDENTIFIED intraoperatively in a patient having a primary TAD, what should be done?

◯ Perform a SNB and remove any palpably abnormal nodes and await histology results (further surgery likely to be needed if no involved nodes are identified on pathology)
◯ Perform an axillary node clearance
◯ Other (please give details)

Other intraoperative management of patient in whom clip/localisation device cannot be identified

If NEITHER the SENTINEL NODE(S) NOR the LOCALISED NODE can be identified intraoperatively, what should be done

◯ Perform a 4 node sample including removal of any palpably abnormal nodes
◯ Perform an axillary node clearance
◯ Other (please give details)

Please give details of other management if neither the sentinel node or the localised node can be identified intraoperatively

Intra-operative findings are highly suspicious for UNANTICIPATED extensive disease in a patient randomised to PRIMARY TAD?

◯ Remove localised node, sentinel node and any palpably abnormal nodes and await histology (patient may require further surgery)
◯ Perform an axillary node clearance
◯ Other (please give details)

Other intraoperative management of patient having PRIMARY TAD wound to have UNANTICIPATED extensive disease?

Are there any other potentially difficult situations that we may encounter when performing TAD that require management guidance?

◯ Yes ◯ No

Please give details of potential difficult situation and recommended management

HOW SHOULD AN AXILLARY NODE CLEARANCE (ANC) BE DEFINED IN THE TADPOLE TRIAL?

We also want to make sure that surgeons are performing ANC in a standard way in the TADPOLE trial. This is important so that the comparison between primary TAD and ANC is valid.

Below is a list of the component steps of the ANC procedure identified from the literature and expert opinion.

For each step, please indicate whether you feel that it should be:

i) MANDATORY - i.e. MUST be performed in ALL patients having ANC in the TADPOLE trial
ii) OPTIONAL - i.e. up to individual surgeons whether this step should be done/not done as part of the procedure
iii) PROHIBITED - i.e. MUST NOT be performed in patients having ANC in the TADPOLE trial

in the TADPOLE population - i.e. those with low volume nodal disease (cN0 with 1-2 involved nodes identified on USS)

If you tick that a step is MANDATORY you will be asked to answer some questions about how it should be delivered during the trial (ie. ranging from completely standardised to completely flexible).

	Mandatory	Optional	Prohibited
The axillary vein and other important structures are seen and preserved during the procedure	◯	◯	◯
A level 1 and 2 clearance is performed	◯	◯	◯
A level 3 clearance is routinely performed in the ABSENCE OF PALPABLE DISEASE	◯	◯	◯
Clips are placed at the upper boundary of the dissection to guide radiotherapy	◯	◯	◯
A drain is used	◯	◯	◯

Are there any additional steps of an ANC that should be MANDATORY in the TADPOLE trial?

◯ Yes ◯ No

Please give details of any additional MANDATORY steps in an ANC

Are there any additional steps/actions that should be PROHIBITED during an ANC in the TADPOLE trial?

◯ Yes ◯ No

Please give details of any additional actions that should be PROHIBITED during an ANC

OPERATIVE DETAILS THAT IT WILL BE IMPORTANT TO COLLECT IN THE TADPOLE TRIAL

We want everyone to be confident that both PRIMARY TAD and ANC were performed consistently in the TADPOLE trial. We will therefore plan to collect some additional information about how the procedures were performed.

How important do you feel that it would be to record the following information about the PRIMARY TAD procedure?

1= NOT essential
9 = ABSOLUTELY essential

	1 Not essential	2	3	4	5	6	7	8	9 Absolutely essential
The number of involved lymph nodes biopsied and clipped (TAD group)	◯	◯	◯	◯	◯	◯	◯	◯	◯
The method of sentinel node localisation used (TAD group)	◯	◯	◯	◯	◯	◯	◯	◯	◯
The method for localising the involved node in the TAD group (e.g. wire, SaviScout etc)	◯	◯	◯	◯	◯	◯	◯	◯	◯
The number of involved lymph nodes localised	◯	◯	◯	◯	◯	◯	◯	◯	◯
The timing of localisation	◯	◯	◯	◯	◯	◯	◯	◯	◯
The total number of lymph nodes removed in the TAD group	◯	◯	◯	◯	◯	◯	◯	◯	◯
Whether the localisation device/clip is identified and removed (TAD group)	◯	◯	◯	◯	◯	◯	◯	◯	◯
Whether the localised node was the sentinel node intraoperatively in the TAD group	◯	◯	◯	◯	◯	◯	◯	◯	◯
The method for confirming removal of the localised node (TAD group)	◯	◯	◯	◯	◯	◯	◯	◯	◯
The duration of the axillary procedure (both groups)	◯	◯	◯	◯	◯	◯	◯	◯	◯

Do you think there is any other important information about the OPERATION performed that should be collected on the operative case report forms?

◯ Yes ◯ No

Please provide details of other information that should be collected on the case report forms

SURGEON EXPERIENCE AND TRAINING

If surgeons have not performed PRIMARY TAD before, how important would it be that they had the following experience and/or training before participating in the TADPOLE Study?

	1 Not essential	2	3	4	5	6	7	8	9 Absolutely essential
Watched TADPOLE training videos and/or attended TADPOLE webinar	◯	◯	◯	◯	◯	◯	◯	◯	◯
Were familiar with performing TAD in patients following neoadjuvant treatment and had performed a pre-specified number of cases	◯	◯	◯	◯	◯	◯	◯	◯	◯
Were participating in the ATNEC study	◯	◯	◯	◯	◯	◯	◯	◯	◯

How many TAD procedures do you think a surgeon should have performed before they can participate in the TADPOLE trial?

◯ At least 5 ◯ 6-10 ◯ More than 10

Is there any other training/experience that you feel would be important before surgeons are able to perform PRIMARY TAD in the TADPOLE trial?

CITABLE COLLABORATOR STATUS

If you would like to be named as a citable collaborator on the future publication, please also provide your first and last name and institution as you would like them to appear in the publication*
*Only individuals who complete BOTH rounds of the survey will meet the criteria for citable collaborator status

First Name ______________________

Surname/Family name ______________________

Name of Institution ______________________

IN PERSON CONSENSUS MEETING, EXPRESSION OF INTEREST
Bristol 20th January 2025

The final stage of this project is an in-person Consensus Meeting at which the approach to axillary surgery in the TADPOLE trial will be discussed and agreed.

The Consensus Meeting will be held in Bristol on 20th January 2025.

If you are interested in participating in the meeting, please complete the following section. Travel expenses will be reimbursed.

Would you be interested in attending the in person consensus meeting to be held in Bristol on Monday 20th January 2025?	◯ Yes ◯ No
Please provide your full name	__________________________________
Please provide the most appropriate e-mail address so we are able to contact you about the meeting (this will not be used for any other reason)	__________________________________

Thank you for expressing an interest in attending the Consensus Meeting!
We will be in touch shortly with further details.

Thank you very much for completing the survey! Your time and expertise is greatly appreciated.

We will be in contact in the next 6-8 weeks with the 2nd Survey.

TADPOLE TOGETHER Round 2 Survey

TADPOLE-TOGETHER

Thank you very much for participating in Round 1 of the TADPOLE TOGETHER survey.

Over 200 surgeons who regularly perform TAD completed the survey and gave their views about how TAD and ANC should be performed in the TADPOLE trial.

We would now be extremely grateful if you could complete the Round 2 survey.

This survey contains feedback on the views of other surgeons completing Round 1 as well as your views from Round 1. We would like you to review your scores and re-score each item in light of the feedback received. You can keep the same score of change.

When scoring, please keep in mind that the aim of this survey is to generate CONSENSUS - agreement about how things should be done in the TADPOLE study.

If you think something is ESSENTIAL - please score it 9

If you think something is NOT ESSENTIAL - please score it a 1

Also think carefully about whether you think something MUST BE STANDARDISED within the trial. This means that it will need to be done in the SAME WAY in ALL 40 + CENTRES PARTICIPATING IN THE TADPOLE TRIAL as well as potential centres in Europe and Australia. Consider whether this is absolutely necessary. We know that many things are IMPORTANT TO REPORT (e.g. whether a clip or a SCOUT is used to localise the node) but TADPOLE is a pragmatic trial so we need to give surgeons and centres some flexibility about how things are done while ensuring TAD and ANC are performed in a consistent way within the trial.

Anyone who completes both rounds of the Delphi survey will be PUBMED citable on the final TADPOLE TOGETHER paper.

If you expressed an interest about coming to the consensus workshop in Bristol on 20th January 2025, we will be in touch separately.

Thank you again for helping us agree how TAD should be done in TADPOLE! We couldn't do this work without your input.

The TADPOLE-TOGETHER Team

HOW SHOULD A PRIMARY TAD BE PERFORMED?

The following is a list of the component steps of a PRIMARY TAD procedure identified from the literature and expert opinion from the Round 1 Survey.

In Round 1 participating surgeons rated each step as:

i) MANDATORY - i.e. MUST be performed in ALL cases of TAD in the TADPOLE trial
ii) OPTIONAL - i.e. up to individual surgeons whether this step should be done/not done as part of the procedure
iii) PROHIBITED - i.e. MUST NOT be performed in TAD patients

Each component of PRIMARY TAD is presented again below with feedback about the percentage of surgeons rating the component as mandatory, optional or prohibited in Round 1 together with your own score.

Please re-score each item in light of the feedback received.

The involved lymph node is localised prior to surgery
ROUND 1 RESPONSES

YOUR RESPONSE FROM ROUND 1 - [localise_mop:label]

Based on the feedback above, when performing PRIMARY TAD should localisation of the involved lymph node before surgery be:

◯ Mandatory ◯ Optional ◯ Prohibited

There is confirmation that the involved node is correctly localised prior to surgery

ROUND 1 RESPONSES

YOUR RESPONSE FROM ROUND 1 - [confirm_mop:label]

Based on feedback above, when performing PRIMARY TAD should confirmation that the involved lymph node is correctly localised prior to surgery be:

◯ Mandatory ◯ Optional ◯ Prohibited

The skin is marked to demonstrate the position of the localised node prior to surgery
ROUND 1 RESPONSE

YOUR RESPONSE FROM ROUND 1 - [skin_mark_mop:label]

Based on the feedback above, when performing PRIMARY TAD, should MARKING the SKIN to demonstrate the position of the localised node prior to surgery be:

◯ Mandatory ◯ Optional ◯ Prohibited

A sentinel node biopsy is performed as part of the primary TAD procedure

ROUND 1 RESPONSES

YOUR RESPONSE FROM ROUND 1 - [snb_mop:label]

Based on the feedback above, when performing PRIMARY TAD, should the performance of sentinel lymph node biopsy as part of the TAD procedure be:

◯ Mandatory ◯ Optional ◯ Prohibited

The localised node is removed during the TAD procedure
ROUND 1 RESPONSES

YOUR ROUND 1 RESPONSE - [remove_node_mop:label]

Based on the feedback above, when performing a PRIMARY TAD, should the removal of the localised node during the TAD procedure be:

◯ Mandatory ◯ Optional ◯ Prohibited

Removal of the localised node is confirmed intra-operatively
ROUND 1 RESPONSES

YOUR ROUND 1 RESPONSE - [remove_conform_mop:label]

Based on the feedback above, when performing a PRIMARY TAD, should intra-operative confirmation of the removal of localised node be:

◯ Mandatory ◯ Optional ◯ Prohibited

All sentinel nodes are removed during the primary TAD procedure
ROUND 1 RESPONSES

YOUR ROUND 1 RESPONSE - [snb_nodes_mop:label]

Based on the feedback above, when performing a PRIMARY TAD, should the removal of all sentinel nodes during the primary TAD procedure be:

◯ Mandatory ◯ Optional ◯ Prohibited

Any palpably ABNORMAL lymph nodes are removed during the primary TAD procedure

ROUND 1 RESPONSES

YOUR ROUND 1 RESPONSE - [palp_nodes_mop:label]

Based on the feedback above, when performing a primary TAD, should the removal of any palpably ABNORMAL lymph nodes be:

◯ Mandatory ◯ Optional ◯ Prohibited

A specified MINIMUM number of nodes are removed during primary TAD procedure

ROUND 1 RESPONSES

YOUR ROUND 1 RESPONSE - [node_no_mop:label]

Based on the feedback above, when performing a PRIMARY TAD, should the removal of a specified MINIMUM number of nodes during the TAD procedure be:

◯ Mandatory ◯ Optional ◯ Prohibited

The TOTAL NUMBER of lymph nodes that can be removed in the TAD group is limited

ROUND 1 RESPONSES

YOUR ROUND 1 RESPONSE - [total_node_mop:label]

Based on the feedback above, when perfroming a PRIMARY TAD, a limit on the TOTAL NUMBER of lymph nodes that can be removed should be:

◯ Mandatory ◯ Optional ◯ Prohibited

Additional NORMAL non-sentinel lymph nodes (not including localised and/or sentinel nodes) are removed

ROUND 1 RESPONSES

YOUR ROUND 1 RESPONSE - [other_nodes_mop:label]

Based on the feedback above, when performing a PRIMARY TAD, should the removal of additional NORMAL non-sentinel lymph nodes (not including localised and/or sentinel nodes) be:

◯ Mandatory ◯ Optional ◯ Prohibited

A drain is inserted during the procedure
ROUND 1 RESPONSES

YOUR ROUND 1 RESPONSE - [drain_mop:label]

Based on the feedback above, when performing a PRIMARY TAD, the insertion of a drain should be:

◯ Mandatory ◯ Optional ◯ Prohibited

Local anaesthetic is used during the procedure
ROUND 1 RESPONSES

YOUR ROUND 1 RESPONSE - [la_mop:label]

Based on the feedback above, when performing a PRIMARY TAD, the use of local anaesthetic during the procedure should be:

◯ Mandatory ◯ Optional ◯ Prohibited

LOCALISING THE INVOLVED NODE PRIOR TO SURGERY

How essential is it that the METHOD for localising the node is STANDARDISED (i.e. MUST be done in the SAME WAY in ALL units participating in the TADPOLE trial).

Median score from Round 1 Surgeons = 6

Your score from Round 1 was [method:value]

Based on the feedback above, how essential is it that the METHOD for localising the node is standardarised (i.e. must be done in the same way in ALL participating units) in the TADPOLE trial?

◯ 1 Not essential ◯ 2 ◯ 3 ◯ 4 ◯ 5 ◯ 6 ◯ 7 ◯ 8 ◯ 9 Absolutely essential

---

What method for localising the involved node should be mandated in the trial?

ROUND 1 RESPONSE

Based on the feedback received, what METHOD FOR LOCALISING THE INVOLVED NODE should be mandated in the trial?

◯ Localisation device (MagSeed, SaviScout, Pintuition, iodine seed etc)
◯ Clip and wire localisation
◯ Carbon tattooing
◯ Method of localising the involved node should be as per local unit practice

---

How essential is it that the TIMING of localisation is STANDARDISED (i.e. MUST be done in the SAME WAY in ALL units participating in the TADPOLE trial).

Median score from Round 1 Surgeons= 5

Your score from Round 1 was [timing:value]

Based on the feedback above, how essential is it that the TIMING of localisation is standardarised (i.e. must be done in the same way in ALL participating units) in the TADPOLE trial?

◯ 1 Not essential ◯ 2 ◯ 3 ◯ 4 ◯ 5 ◯ 6 ◯ 7 ◯ 8 ◯ 9 Absolutely essential

---

If it is necessary to standardise the TIMING of the LOCALISATION of the involved node, when should this be?

ROUND 1 RESPONSE

Based on the feedback received, when if the localisation of the involved node is to be standardised, what should the TIMING of the localisation be?

◯ At the time of biopsy (before nodal involvement confirmed)
◯ At any time after nodal involvement is confirmed
◯ On the day of surgery
◯ As per local unit practice

CONFIRMATION that the INVOLVED LYMPH NODE has been CORRECTLY LOCALISED prior to surgery

How essential is it that the method for HOW we CONFIRM THAT THE INVOLVED NODE has been CORRECTLY LOCALISED (e.g US v signal from tag) PRIOR TO SURGERY is STANDARDISED (i.e. MUST be done in the SAME WAY in ALL units participating in the TADPOLE trial).

Median score from Round 1 Surgeons = 7

Your score from Round 1 was [confirm_how:value]

Based on the feedback above, how essential is it that the METHOD for HOW we CONFIRM THAT THE INVOLVED NODE has been CORRECTLY LOCALISED PRIOR TO SURGERY is standardised (i.e. must be done in the same way in ALL participating units) in the TADPOLE trial?

◯ 1 Not essential ◯ 2 ◯ 3 ◯ 4 ◯ 5 ◯ 6 ◯ 7 ◯ 8 ◯ 9 Absolutely essential

---

HOW should confirmation of localisation be mandated in the trial?

RESULTS FROM ROUND 1

Based on the feedback above, HOW should the CONFIRMATION of the LOCALISATION OF THE INVOLVED NODE PRIOR TO SURGERY be mandated in the trial?

◯ As per local unit practice
◯ By detecting a signal if localisation device is used
◯ Using USS following placement of wire/localisation device

How essential is it that WHEN the CONFIRMATION THAT THE INVOLVED NODE has been CORRECTLY LOCALISED (e.g US v signal from tag) PRIOR TO SURGERY is STANDARDISED (i.e. MUST be done in the SAME WAY in ALL units participating in the TADPOLE trial).

Median score from Round 1 Surgeons = 7

Your score from Round 1 = [confirm_when:value]

Based on the feedback above, how essential is it that WHEN the CONFIRMATION THAT THE INVOLVED NODE has been CORRECTLY LOCALISED (e.g US v signal from tag) PRIOR TO SURGERY is STANDARDISED (i.e. MUST be done in the SAME WAY in ALL units participating in the TADPOLE trial)?

◯ 1 Not essential ◯ 2 ◯ 3 ◯ 4 ◯ 5 ◯ 6 ◯ 7 ◯ 8 ◯ 9 Absolutely essential

---

WHEN should confirmation of localisation be performed?

RESULTS FROM ROUND 1

Based on the feedback above, WHEN should the CONFIRMATION OF LOCALISATION of the INVOLVED NODE be MANDATED in the trial?

◯ As per local unit practice
◯ At the point of wire/localisation device insertion
◯ On the day of surgery before patient is anaesthetised
◯ Both time points

MARKING the SKIN to demonstrate the position of the localised node prior to surgery

How essential is it to standardise HOW the SKIN is MARKED to demonstrate the position of the localised node prior to surgery (i.e. must be done in the SAME WAY in ALL units participating in the TADPOLE trial)?

Median score from Round 1 Surgeons = 7

Your score from Round 1 = [skin_how_standard:value]

Based on the feedback above, how essential is it that HOW the SKIN is MARKED (e.g. USS vs detection of signal from tag) to demonstrate the position of the localised node prior to surgery is STANDARDISED?

◯ 1 Not essential ◯ 2 ◯ 3 ◯ 4 ◯ 5 ◯ 6 ◯ 7 ◯ 8 ◯ 9 Absolutely essential

How essential is it to standardise WHEN the SKIN is MARKED to demonstrate the position of the localised node prior to surgery (i.e. must be done in the SAME WAY in ALL units participating in the TADPOLE trial)?

Median score from Round 1 Surgeons = 7

Your score from Round 1 = [skin_when_standard:value]

Based on the feedback above, how essential is it that WHEN the SKIN is MARKED to demonstrate the position of the localised node prior to surgery is STANDARDISED?

◯ 1 Not essential ◯ 2 ◯ 3 ◯ 4 ◯ 5 ◯ 6 ◯ 7 ◯ 8 ◯ 9 Absolutely essential

Performing a SENTINEL NODE BIOPSY is a MANDATORY step in a primary TAD procedure

If performing a sentinel node biopsy as part of the primary TAD procedure is MANDATORY, how essential is it that the sentinel node localisation is performed in a standardised way rather than as per local unit policy in the trial?

Results from Round 1 Surgeons = 7

Your score from Round 1 = [sn_loc_stand:value]

Based on the feedback above, how essential is it to standarise the performance of SENTINEL NODE LOCALISATION so that it is performed in the same way in ALL units in the TADPOLE trial, rather than as per local unit policy?

○ 1 Not essential ○ 2 ○ 3 ○ 4 ○ 5 ○ 6 ○ 7 ○ 8 ○ 9 Absolutely essential

---

If it is absolutely essential that the sentinel node localisation is performed in a standardised way, how should it be performed?

Based on the feedback above, if SENTINEL NODE LOCALISATION is performed in a standardised way, how should it be performed across ALL sites in the TADPOLE trial?

○ As per local unit practice
○ Dual tracer technique (blue dye and radioactive isotope)
○ Single tracer (radioactive isotope)
○ Single tracer (any)

If isotope is used as part of the sentinel node localisation, how essential is it that lymphoscintography is performed?

Median score from Round 1 Surgeons = 1

Your score from Round 1 = [lymphoscint:value]

Based on the feedback above, if ISOTOPE is used as part of the sentinel node localisation, how essential is that lymphoscintography is performed as standard, across ALL sites in the TADPOLE trial?

○ 1 Not essential ○ 2 ○ 3 ○ 4 ○ 5 ○ 6 ○ 7 ○ 8 ○ 9 Absolutely essential

---

If performing a sentinel node biopsy as part of the primary TAD procedure is MANDATORY, how essential is it that the POSITION of the sentinel node localisation INJECTION in the breast is standardised in the trial?

Median score from Round 1 Surgeons = 5

Your score from Round 1 = [local_inj:value]

Based on the feedback above, how essential is it that that the POSITION of the sentinel node localisation INJECTION in the breast is STANDARISED in the trial (i.e. the same position of localisation injection is used in ALL sites across the TADPOLE trial)?

○ 1 Not essential ○ 2 ○ 3 ○ 4 ○ 5 ○ 6 ○ 7 ○ 8 ○ 9 Absolutely essential

---

What position should the localisation injection be located?

RESULTS FROM ROUND 1

Based on the feedback above, what POSITION for SENTINEL NODE LOCALISATION INJECTION should be MANDATED in the trial?

○ As per local unit practice
○ In the same quadrant as the cancer
○ At the 12 o'clock position
○ Peri or subareolar

How essential is it that the SNB is performed BEFORE removal of the LOCALISED node?

Median score from Round 1 Surgeons = 1

Your score from Round 1 = [snb_first:value]

Based on the feedback above, how essential is it to MANDATE that the SNB is performed BEFORE removal of the LOCALISED node in the TADPOLE trial?

◯ 1 Not essential ◯ 2 ◯ 3 ◯ 4 ◯ 5 ◯ 6 ◯ 7 ◯ 8 ◯ 9 Absolutely essential

Numbers of lymph nodes removed in a PRIMARY TAD and confirmation of removal of the localised node

If intra-operative confirmation of removal of the localised involved lymph node is MANDATORY, how essential is it that confirmation is done in a standardised way within the trial (e.g intraoperative radiograph vs detection of signal from in removed node)?

Median score from Round 1 Surgeons = 8

Your score from Round 1 = [removal_confirm_stand:value]

Based on the feedback above, if intra-operative confirmation of removal of the localised involved lymph node is MANDATORY, how essential is it that CONFIRMATION is done in a STANDARDISED way (i.e. the same technique used at ALL sites in the trial e.g intraoperative radiograph vs detection of signal from removed node)?

◯ 1 Not essential ◯ 2 ◯ 3 ◯ 4 ◯ 5 ◯ 6 ◯ 7 ◯ 8 ◯ 9 Absolutely essential

---

If removal of a specified MINIMUM number of nodes during a primary TAD procedure is MANDATORY, how many lymph nodes should this be?

RESULTS FROM ROUND 1

Based on the feedback above if removal of a specified MINIMUM number of nodes during a primary TAD procedure is MANDATORY, how many lymph nodes should this be?

◯ 1
◯ 2
◯ 3
◯ 4
◯ No pre-specified number of nodes as long as targeted and sentinel nodes are removed

---

If TOTAL NUMBER of lymph nodes that can be removed in patients having PRIMARY TAD is limited, what should be the MAXIMUM number of nodes that can be removed?

RESULTS FROM ROUND 1

Based on the feedback above, if TOTAL NUMBER of lymph nodes that can be removed in patients having PRIMARY TAD is limited, what should be the MAXIMUM number of nodes that can be removed?

◯ 3
◯ 4
◯ 5
◯ 6
◯ 7
◯ No maximum number of nodes should be mandated

MANAGEMENT OF DIFFICULT SITUATIONS IN PATIENTS HAVING PRIMARY TAD

We would like to establish consensus about how best to manage potential problems that may be encountered when performing a PRIMARY TAD.

Please consider the scenarios below and select what option you feel would be best in each one.

If the sentinel node localisation technique FAILS (e.g. no blue dye/isotope in the axilla) in a patient having a PRIMARY TAD, what would you consider the best course of action intraoperatively?

RESULTS FROM ROUND 1

YOUR ROUND 1 RESPONSE - [failed_snb:label]

Based on the feedback above, if the sentinel node localisation technique FAILS (e.g. no blue dye/isotope in the axilla) in a patient having a PRIMARY TAD, what would you consider the best course of action intraoperatively?

◯ Remove the localised involved node and perform a 4 node sample (including any palpably abnormal nodes)
◯ Perform an axillary node clearance

If the clip/localisation device is identified, but it is NOT in the involved node in a patient having a PRIMARY TAD, what should be done intraoperatively?

RESULTS FROM ROUND 1

YOUR ROUND 1 RESPONSE - [clipoutsidenode:label]

Based on the feedback above, if the clip/localisation device is identified, but it is NOT in the involved node in a patient having a PRIMARY TAD, what should be done intraoperatively?

◯ Perform the sentinel node biopsy and remove any palpably abnormal nodes and nothing else
◯ Perform the SNB, remove any palpably abnormal nodes AND any nodes close to the clip/localisation device
◯ Perform an axillary node clearance

If the clip/localisation device CANNOT BE IDENTIFIED intraoperatively in a patient having a primary TAD, what should be done?

RESULTS FROM ROUND 1

YOUR ROUND 1 RESPONSE - [nolocaliser_clip:label]

Based on the feedback above, if the clip/localisation device CANNOT BE IDENTIFIED intraoperatively in a patient having a primary TAD, what should be done?

◯ Perform a SNB and remove any palpably abnormal nodes and await histology results (further surgery likely to be needed if no involved nodes are identified on pathology)
◯ Perform an axillary node clearance

If NEITHER the SENTINEL NODE(S) NOR the LOCALISED NODE can be identified intraoperatively, what should be done

RESULTS FROM ROUND 1

YOUR ROUND 1 RESPONSE - [nosnborloc:label]

Based on the feedback above, if NEITHER the SENTINEL NODE(S) NOR the LOCALISED NODE can be identified intraoperatively, what should be done

○ Perform a 4 node sample including removal of any palpably abnormal nodes
○ Perform an axillary node clearance

Intra-operative findings are highly suspicious for UNANTICIPATED extensive disease in a patient randomised to PRIMARY TAD?

RESULTS FROM ROUND 1

YOUR ROUND 1 RESPONSE - [extensive_disease:label]

Based on the above feedback, how do you manage a scenario where intra-operative findings are highly suspicious for UNANTICIPATED extensive disease in a patient randomised to PRIMARY TAD?

○ Remove localised node, sentinel node and any palpably abnormal nodes and await histology (patient may require further surgery)
○ Perform an axillary node clearance

HOW SHOULD AN AXILLARY NODE CLEARANCE (ANC) BE DEFINED IN THE TADPOLE TRIAL?

We also want to make sure that surgeons are performing ANC in a standard way in the TADPOLE trial. This is important so that the comparison between primary TAD and ANC is valid.

Below is a list of the component steps of the ANC procedure identified from the literature and expert opinion.

For each step, please indicate whether you feel that it should be:

i) MANDATORY - i.e. MUST be performed in ALL patients having ANC in the TADPOLE trial
ii) OPTIONAL - i.e. up to individual surgeons whether this step should be done/not done as part of the procedure
iii) PROHIBITED - i.e. MUST NOT be performed in patients having ANC in the TADPOLE trial

in the TADPOLE population - i.e. those with low volume nodal disease (cN0 with 1-2 involved nodes identified on USS)

If you tick that a step is MANDATORY you will be asked to answer some questions about how it should be delivered during the trial (ie. ranging from completely standardised to completely flexible).

The axillary vein and other important structures are seen and preserved during the procedure

RESULTS FROM ROUND 1

Your Round 1 Response - [structures_mop:label]

Based on the feedback above, when performing an ANC as part of the TADPOLE TRIAL should the identification and preservation of the axillary vein and other important structures be:

◯ Mandatory ◯ Optional ◯ Prohibited

A level 1 and 2 clearance is performed

RESULTS FROM ROUND 1

Your Round 1 Response - [level1and2_mop:label]

Based on the feedback above, when performing an ANC as part of the TADPOLE trial, should the performance of a level 1 and 2 clearance be:

◯ Mandatory ◯ Optional ◯ Prohibited

A level 3 clearance is routinely performed in the ABSENCE OF PALPABLE DISEASE

RESULTS FROM ROUND 1

YOUR ROUND 1 RESPONSE - [level3_mop:label]

Based on the above feedback, in ANC performed in the TADPOLE trial, should the routine performance of a level 3 clearance IN THE ABSENCE OF PALPABLE DISEASE be:

◯ Mandatory ◯ Optional ◯ Prohibited

Clips are placed at the upper boundary of the dissection to guide radiotherapy

RESULTS FROM ROUND 1

YOUR ROUND 1 RESPONSE - [clips_mop:label]

Based on the feedback above, when performing an ANC as part of the TADPOLE trial, should placement of clips at the upper boundary of dissection to guide radiotherapy be:

◯ Mandatory ◯ Optional ◯ Prohibited

A drain is used

RESULTS FROM ROUND 1

YOUR ROUND 1 RESPONSE - [draining_mop:label]

Based on the above feedback, when performing an ANC in the TADPOLE trial, should the use of a drain be:

◯ Mandatory ◯ Optional ◯ Prohibited

OPERATIVE DETAILS THAT IT WILL BE IMPORTANT TO COLLECT IN THE TADPOLE TRIAL

We want everyone to be confident that both PRIMARY TAD and ANC were performed consistently in the TADPOLE trial. We will therefore plan to collect some additional information about how the procedures were performed.

How important do you feel that it would be to record the following information about the PRIMARY TAD procedure?

1= NOT essential
9 = ABSOLUTELY essential

The number of involved lymph nodes biopsied and clipped (TAD group)

Median score from Round 1 Surgeons = 9

Your score from Round 1 = [no_nodes_clipped:value]

Based on the feedback above, how essential is it to record the number of involved lymph nodes biopsied and clipped (TAD group)?

◯ 1 Not essential ◯ 2 ◯ 3 ◯ 4 ◯ 5 ◯ 6 ◯ 7 ◯ 8 ◯ 9 Absolutely essential

---

The method of sentinel node localisation used (TAD group)

Median score from round 1 Surgeons = 9

Your score from Round 1 = [snb_method:value]

Based on the feedback above, how essential is it to record the method of sentinel node localisation used (TAD group)?

◯ 1 Not essential ◯ 2 ◯ 3 ◯ 4 ◯ 5 ◯ 6 ◯ 7 ◯ 8 ◯ 9 Absolutely essential

---

The method for localising the involved node in the TAD group (e.g. wire, SaviScout etc)

Median score from Round 1 Surgeons = 9

Your score from Round 1 = [loc_method:value]

Based on the feedback above, how essential is it to record the method for localising the involved node in the TAD group (e.g. wire, SaviScout etc)

◯ 1 Not essential ◯ 2 ◯ 3 ◯ 4 ◯ 5 ◯ 6 ◯ 7 ◯ 8 ◯ 9 Absolutely essential

The number of involved lymph nodes localised

Median score from Round 1 Surgeons = 9

Your score from Round 1 = [no_nodes_loc:value]

Based on the feedback above, how essential is it to record the number of involved lymph nodes localised?

○ 1 Not essential ○ 2 ○ 3 ○ 4 ○ 5 ○ 6 ○ 7 ○ 8 ○ 9 Absolutely essential

---

The timing of localisation

Median score from Round 1 Surgeons = 5

Your score from Round 1 = [time_loc:value]

Based on the feedback above, how essential is it to record the timing of localisation?

○ 1 Not essential ○ 2 ○ 3 ○ 4 ○ 5 ○ 6 ○ 7 ○ 8 ○ 9 Absolutely essential

---

The total number of lymph nodes removed in the TAD group

Median score from Round 1 = 9

Your score from Round 1 = [total_nodes:value]

Based on the feedback above, how essential is it to record the number of lymph nodes removed in the TAD group?

○ 1 Not essential ○ 2 ○ 3 ○ 4 ○ 5 ○ 6 ○ 7 ○ 8 ○ 9 Absolutely essential

---

Whether the localisation device/clip is identified and removed (TAD group)

Median score from Round 1 Surgeons = 9

Your score from Round 1 = [device_removed:value]

Based on the feedback above, how essential is it to record whether the localisation device/clip is identified and removed (TAD group)?

○ 1 Not essential ○ 2 ○ 3 ○ 4 ○ 5 ○ 6 ○ 7 ○ 8 ○ 9 Absolutely essential

---

Whether the localised node was the sentinel node intraoperatively in the TAD group

Median score from Round 1 = 9

Your score from Round 1 = [loc_snb:value]

Based on the feedback above, how essential is it to record whether the localised node was the sentinel node intraoperatively in the TAD group

○ 1 Not essential ○ 2 ○ 3 ○ 4 ○ 5 ○ 6 ○ 7 ○ 8 ○ 9 Absolutely essential

The method for confirming removal of the localised node (TAD group)

Median score from Round 1 Surgeons = 8

Your score from Round 1 = [confirm_removal:value]

Based on the feedback above, how essential is it to record the method for confirming removal of the localised node (TAD group)

◯ 1 Not essential ◯ 2 ◯ 3 ◯ 4 ◯ 5 ◯ 6 ◯ 7 ◯ 8 ◯ 9 Absolutely essential

---

The duration of the axillary procedure (both groups)

Median score from Round 1 = 5

Your score from Round 1 = [duration:value]

Based on the feedback above, how essential is it to record the duration of the axillary procedure (both groups)

◯ 1 Not essential ◯ 2 ◯ 3 ◯ 4 ◯ 5 ◯ 6 ◯ 7 ◯ 8 ◯ 9 Absolutely essential

SURGEON EXPERIENCE AND TRAINING

If surgeons have not performed PRIMARY TAD before, how important would it be that they had the following experience and/or training before participating in the TADPOLE Study?

Watched TADPOLE training videos and/or attended TADPOLE webinar

Median score from Round 1 = 8

Your score from Round 1 = [training:value]

Based on the feedback above, how essential is it that surgeons involved in the TADPOLE trial have watched TADPOLE training videos and/or attended TADPOLE webinar

◯ 1 Not essential ◯ 2 ◯ 3 ◯ 4 ◯ 5 ◯ 6 ◯ 7 ◯ 8 ◯ 9 Absolutely essential

---

Were familiar with performing TAD in patients following neoadjuvant treatment and had performed a pre-specified number of cases

Median score from Round 1 Surgeons = 7

Your score from Round 1 = [nact:value]

Based on the feedback above, how essential is it that surgeons participating in the TADPOLE trial were performing TAD in patients following neoadjuvant treatment and had performed a pre-specified number of cases.

◯ 1 Not essential ◯ 2 ◯ 3 ◯ 4 ◯ 5 ◯ 6 ◯ 7 ◯ 8 ◯ 9 Absolutely essential

---

Were participating in the ATNEC study

Median score from Round 1 Surgeons = 3

Your score from Round 1 = [atnec:value]

Based on the feedback above, how essential is it that surgeons participating in the TADPOLE trial were participating in the ATNEC study.

◯ 1 Not essential ◯ 2 ◯ 3 ◯ 4 ◯ 5 ◯ 6 ◯ 7 ◯ 8 ◯ 9 Absolutely essential

---

How many TAD procedures do you think a surgeon should have performed before they can participate in the TADPOLE trial?

RESULTS FROM ROUND 1

Based on the feedback above, how many TAD procedures do you think a surgeon should have performed before they can participate in the TADPOLE trial?

◯ No pre-specified minimum number of TAD procedures completed ◯ More than 10 ◯ 6 - 10
◯ At least 5

CITABLE COLLABORATOR STATUS

If you would like to be named as a citable collaborator on the future publication, please also provide your first and last name and institution as you would like them to appear in the publication*
*Only individuals who complete BOTH rounds of the survey will meet the criteria for citable collaborator status

First Name ________________________________

Surname/Family name ________________________________

Name of Institution ________________________________

Thank you very much for completing the survey! Your time and expertise is greatly appreciated.

EOI Virtual meeting

We are now planning to hold the TADPOLE-TOGETHER Consensus Meeting VIRTUALLY between 1.30 and 4.30pm on Monday 20th January.

Please indicate if you would be able to attend below.

Many thanks in advance

Shelley Potter

Lead Investigator for the TADPOLE study

---

158) I will be able to attend AN ONLINE CONSENSUS MEETING between 1.30 and 4.30 on Monday 20th January ○ Yes ○ No
